# Felodipine induces autophagy in mouse brains with pharmacokinetics amenable to repurposing

**DOI:** 10.1038/s41467-019-09494-2

**Published:** 2019-04-18

**Authors:** Farah H. Siddiqi, Fiona M. Menzies, Ana Lopez, Eleanna Stamatakou, Cansu Karabiyik, Rodrigo Ureshino, Thomas Ricketts, Maria Jimenez-Sanchez, Miguel Angel Esteban, Liangxue Lai, Micky D. Tortorella, Zhiwei Luo, Hao Liu, Emmanouil Metzakopian, Hugo J. R. Fernandes, Andrew Bassett, Eric Karran, Bruce L. Miller, Angeleen Fleming, David C. Rubinsztein

**Affiliations:** 1 Department of Medical Genetics, Cambridge Institute for Medical Research, University of Cambridge, The Keith Peters Building, Cambridge Biomedical Campus, Hills Road, Cambridge, CB2 0XY UK; 2 UK Dementia Research Institute, Cambridge Institute for Medical Research, University of Cambridge, The Keith Peters Building, Cambridge Biomedical Campus, Hills Road, Cambridge, CB2 0XY UK; 3 Department of Physiology, Development and Neuroscience, University of Cambridge, Downing Street, Cambridge, CB2 3DY UK; 4 Guangzhou Institutes of Biomedicine and Health, Chinese Academy of Sciences, 190 Kai Yuan Avenue, Science Park, 501530 Guangzhou, China; 5 UK Dementia Research Institute, Department of Clinical Neurosciences, University of Cambridge, Cambridge, CB2 0AH UK; 6 Wellcome Sanger Institute, Wellcome Genome Campus, Hinxton, Cambridgeshire CB10 1SA UK; 7 AbbVie Inc., Foundational Neuroscience Center, 200 Sidney Street, Cambridge, MA 02139 USA; 8 Memory and Aging Center, Department of Neurology, University of California, San Francisco, CA USA; 9 Present Address: Department of Basic and Clinical Neuroscience, King’s College London, Institute of Psychiatry, Psychology and Neuroscience, Maurice Wohl Clinical Neuroscience Institute, London, SE5 9RX UK

**Keywords:** Pharmacology, Pharmacology, Target validation, Target validation, Neurological disorders

## Abstract

Neurodegenerative diseases like Alzheimer’s disease, Parkinson’s disease and Huntington’s disease manifest with the neuronal accumulation of toxic proteins. Since autophagy upregulation enhances the clearance of such proteins and ameliorates their toxicities in animal models, we and others have sought to re-position/re-profile existing compounds used in humans to identify those that may induce autophagy in the brain. A key challenge with this approach is to assess if any hits identified can induce neuronal autophagy at concentrations that would be seen in humans taking the drug for its conventional indication. Here we report that felodipine, an L-type calcium channel blocker and anti-hypertensive drug, induces autophagy and clears diverse aggregate-prone, neurodegenerative disease-associated proteins. Felodipine can clear mutant α-synuclein in mouse brains at plasma concentrations similar to those that would be seen in humans taking the drug. This is associated with neuroprotection in mice, suggesting the promise of this compound for use in neurodegeneration.

## Abstract (web summary)

A key challenge is to find/re-purpose approved drugs that could be used in humans to induce autophagy-associated clearance of neurodegenerative proteins. Here, authors demonstrate that felodipine, an anti-hypertensive drug, can induce autophagy and clear a variety of aggregated neurodegenerative disease-associated proteins in mouse brains at plasma concentrations similar to those that would be seen in humans taking the drug.

## Introduction

A common feature of most neurodegenerative diseases, including Alzheimer’s, Parkinson’s and Huntington’s disease (HD) is the accumulation of aggregate-prone proteins within the cytoplasm of neurons. Such proteins, like mutant huntingtin (HD), α-synuclein (Parkinson’s disease) and tau (in various dementias), cause pathology via toxic gain-of-function mechanisms. Thus, the factors regulating their clearance are likely to be important for understanding pathogenesis and developing rational therapeutic strategies.

Autophagy, a major intracytoplasmic protein degradation pathway, is mediated by double-membraned structures, which engulf portions of cytoplasm. Autophagosomes ultimately fuse with lysosomes, where their contents are degraded. Autophagy is critical for the degradation of diverse intracytoplasmic aggregate-prone proteins that cause neurodegenerative diseases, including huntingtin, mutant α-synuclein, and tau^1–4^ and impairing autophagy causes the accumulation of such proteins and enhances their toxicities. Autophagy upregulation using compounds or genetic means enhances the clearance of such proteins and thereby attenuates the deleterious phenotypes seen in *Drosophila*, zebrafish and mouse models of these diseases^5^. Hence, we and others have screened existing drugs approved for other indications to identify autophagy inducers that may be suitable for treating neurodegenerative diseases. While these efforts have identified a number of existing drugs as autophagy inducers, the animal studies using such repurposed drugs have not tested whether any benefits that they may have are achievable with concentrations of the compounds that exist in people taking these compounds. This issue is critical, as drugs typically have much shorter half-lives in mice compared to humans, and the concentrations that the drugs may reach (albeit transiently) in mouse studies may be orders of magnitude higher than those seen with human dosing, which aims for prolonged and stable levels. Thus, it is possible that the effects of any such compounds seen in mice may not be achievable in humans, as the benefits may be due to on-target or off-target drug effects at concentrations much higher than those seen in humans. This scenario would make the drugs unsuitable for direct repurposing.

Previously, from a screen of a library enriched in approved drugs, we identified verapamil, an L-type calcium channel blocker, as an inducer of autophagosome formation and validated that L-type calcium channels are an mTOR-independent autophagy target using numerous tools^6^. Verapamil enhanced the clearance of Parkinson’s disease-causing A53T mutant α-synuclein in cell lines and reduced the percentages of cells with aggregates of mutant huntingtin exon 1 in autophagy-competent but not in autophagy-null cells. Furthermore, verapamil rescued mutant huntingtin toxicity in *Drosophila* and zebrafish models of Huntington’s disease, where it also reduced huntingtin aggregate numbers. L-type calcium channel blockers are anti-hypertensive drugs and are widely used in man for long-term therapeutic treatment. Therefore, this family of drugs would be suitable for re-purposing for long-term treatment for neurodegenerative disorders. However, verapamil does not cross the blood–brain barrier (BBB) and is therefore not suitable to this end. Hence, we screened a panel of currently prescribed L-type calcium channel blockers to identify a BBB-penetrant member of this class that showed strong autophagy-inducing effects and had a long half-life in man. We selected felodipine as the most suitable candidate for further assessment and validated its effects in in vivo models of tauopathy, Huntington’s disease and Parkinson’s disease (A53T α-synuclein mutation) to test whether this autophagy-inducing drug had relevance to multiple neurodegenerative diseases. We performed pharmacokinetic analysis to determine the optimal treatment regime in mice to mimic the plasma concentration that the drug reaches in man at currently prescribed doses. Finally, we tested felodipine at this clinically-relevant concentrations.

Our data reveal that felodipine administration in mice at concentrations approximating those seen in humans induces autophagy and reduces levels of neurotoxic proteins, like A53T α-synuclein. These data suggest that this drug may have efficacy in humans with appropriate neurodegenerative diseases that may be ameliorated by autophagy induction.

## Results

### Screening of calcium channel blockers in primary neurons

**Fig. 1:**
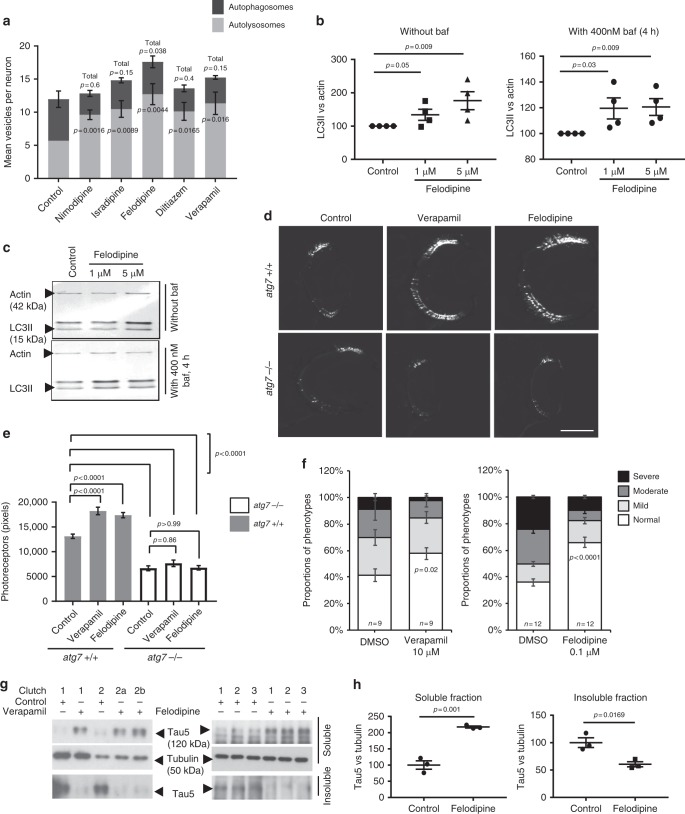
Screening of L-type calcium channel blockers in primary neurons and zebrafish. **a** Quantification of autophagosomes and autolysosomes in primary neurons from mRFP-GFP-LC3 mice treated with L-type calcium channel blockers (at 1 μM). Data represent mean number of vesicles/neuron +/− SEM, 4 independent experiments, two–tailed unpaired *t*-test. *P* values shown for autolysosomes and total vesicle number. **b**, **c** Wild-type primary neurons were treated with 1 and 5 μM felodipine for 5 h with and without 400 nM bafilomycin (last 4 h). Graph shows mean densitometry +/− SEM (LC3II vs. actin; *n* = 4 independent experiments; one-tailed, unpaired *t*-test). Data were normalised to the control (set to 100). **c** Representative western blots for LC3II in wild-type primary cortical neurons. **d**, **e** Verapamil and felodipine were tested in Rho::EGFP-Tau^cu7^ fish—either in autophagy-null (*atg7*^−*/*−^*)* or wild-type-autophagy siblings (*atg7*^*+/+*^). **d** Representative fluorescence images of the GFP-positive rod photoreceptors in sections of Rho::EGFP-Tau^cu7^ fish treated with DMSO, verapamil or felodipine, scale bar = 50 μm. **e** Quantification of rod photoreceptors from images of sections through the central retina after verapamil or felodipine treatment (*n* = 40 eyes/group); one-way ANOVA with post hoc Tukey’s multiple comparison test. Exact *p* values are provided for control (DMSO) compared to drugs for the same genotype. **f** Verapamil and felodipine ameliorated morphological defects in Dendra-tau-A152T fish (see Supplementary Fig. 2a for details of phenotype scoring). Data represent means +/− SEM; *n* ≥ 20 fish/ treatment group from ≥9 clutches. Two-tailed unpaired *t*-test. **g** Verapamil and felodipine increased the levels of sarkosyl-soluble tau and reduced the levels of insoluble tau in fractions from Dendra-tauA152T fish. **h** Graph shows densitometry of the mean ratios (±SEM) of soluble and insoluble tau vs. tubulin from fractions of 3 independent clutches (50 fish/group) normalised to mean DMSO value (set to 100); two-tailed unpaired *t*-test

In order to select the most promising of the L-type calcium channel blockers (Table 1) for further in vivo studies, we researched all currently prescribed FDA-approved members of this family^7^ and ranked them according to BBB-penetration, structural similarity (dihydropyridine or non-dyhydropyridine) and known half-life in man. We selected 5 drugs and profiled their autophagy-inducing activity in primary neurons from a transgenic mouse model expressing mRFP-GFP-LC3^8^. LC3 is recruited to the membrane of forming autophagosomes and is widely accepted to be a reliable marker of autophagic vesicles. When LC3 is double-tagged with both GFP and a red fluorescent protein (e.g., mCherry or mRFP)^9^, one can distinguish non-acidified autophagosomes (red and green = yellow) from acidified autolysosomes (red only), as the GFP fluorescence is more rapidly quenched by the low lysosomal pH^10^. Of the panel of L-type calcium channel blockers tested, felodipine caused the greatest increase on both autophagosome and autolysosome numbers (Fig. 1a and Supplementary Fig. 1a). This was the most sensitive measure of activity since, in mouse primary cortical neurons, all the L-type calcium channel blockers we studied decreased the percentage of neurons with mutant huntingtin exon 1 aggregates to similar extents, a phenomenon seen with other known autophagy inducers, like trehalose and rapamycin (Supplementary Fig. 1b). To verify the effects observed with the mRFP-GFP-LC3 reporter, we confirmed that felodipine increased the steady-state of endogenous LC3-II levels, as well as LC3-II formation (in experiments without and with the lysosome inhibitor, bafilomycin A1) in mouse primary neurons, demonstrating that it increased autophagic flux (Fig. 1b, c). Based on these data and the knowledge that felodipine crosses the BBB, we investigated felodipine further.

**Table 1:** List of selected calcium channel blockers

Drugs	Class	Suggested primary target	Plasma half-life (human)	Crosses BBB
Nimodipine	Dihydropyridine	L-Type channel P/Q channel	1.7–9 h	Yes
Isradipine	Dihydropyridine	L-Type channel	8 h	Yes
Felodipine	Dihydropyridine	L-Type channel T-Type channel	8.5–19.7 h	Yes
Verapamil	Non-dihydropyridine (phenylalkylamine)	L-Type channel B-Type channel P/Q channel	2.8–7.4 h	No
Diltiazem	Non-dihydropyridine (benzothiazepine)	L-Type channel	3–4.5 h	Poor
Amlodipine	Dihydropyridine	L-Type channel	30–50 h	No
Nifedipine	Dihydropyridine	L-Type channel	2–7 h^a^	Yes
Nicardipine	Dihydropyridine	L-Type channel	8.6 h	Yes
Clevidipine	Dihydropyridine	L-Type channel	1 min	Yes
			(2–15 min)	
Nisoldipine	Dihydropyridine	L-Type channel	7–12 h	Yes

The primary target human plasma half-life and BBB penetration information was obtained from PubChem **(**https://pubchem.ncbi.nlm.nih.gov/) and DrugBank (https://www.drugbank.ca/), accurate at the time of manuscript submission

^a^7 h half-life determined from slow release formulation

### L-type calcium channel blockers protect zebrafish models

We next went on to assess the in vivo efficacy of felodipine. We have previously observed that verapamil was beneficial in our zebrafish model of Huntington’s disease^6^. To confirm that L-type channel calcium blockers can clear a range of disease-causing substrates, we compared the efficacy of verapamil and felodipine in two zebrafish transgenic models of tauopathy. The first model (rho::tau) expresses wild-type human tau in the rod photoreceptors which causes progressive degeneration and loss of these cells^11^. Both verapamil and felodipine rescued the degeneration of photoreceptors in these fish but failed to rescue degeneration in rho::tau fish lacking the critical autophagy gene *atg7*, indicating that the rescuing effects are autophagy-dependent (Fig. 1d, e). In the second zebrafish model, pan-neuronal expression of the p.A152T tau variant, associated with dementia and tau pathology in patients^12^ causes tau hyperphosphorylation, tau aggregation and neuronal loss in zebrafish, which are also seen in mouse models^13,14^. Verapamil and felodipine ameliorated the morphological defects in this model (Fig. 1f), defined in supplementary Fig. 2a. Both drugs selectively reduced the levels of insoluble tau and caused an increase in the soluble fractions, suggesting that the insoluble species may be preferentially removed by verapamil or felodipine treatment (Fig. 1g, h). No significant changes in total tau levels were observed suggesting that soluble tau may be sequestered in the insoluble fraction in the DMSO-treated group (Fig. 1g and Supplementary Fig. 2b, c, d, and e).

**Fig. 2:**
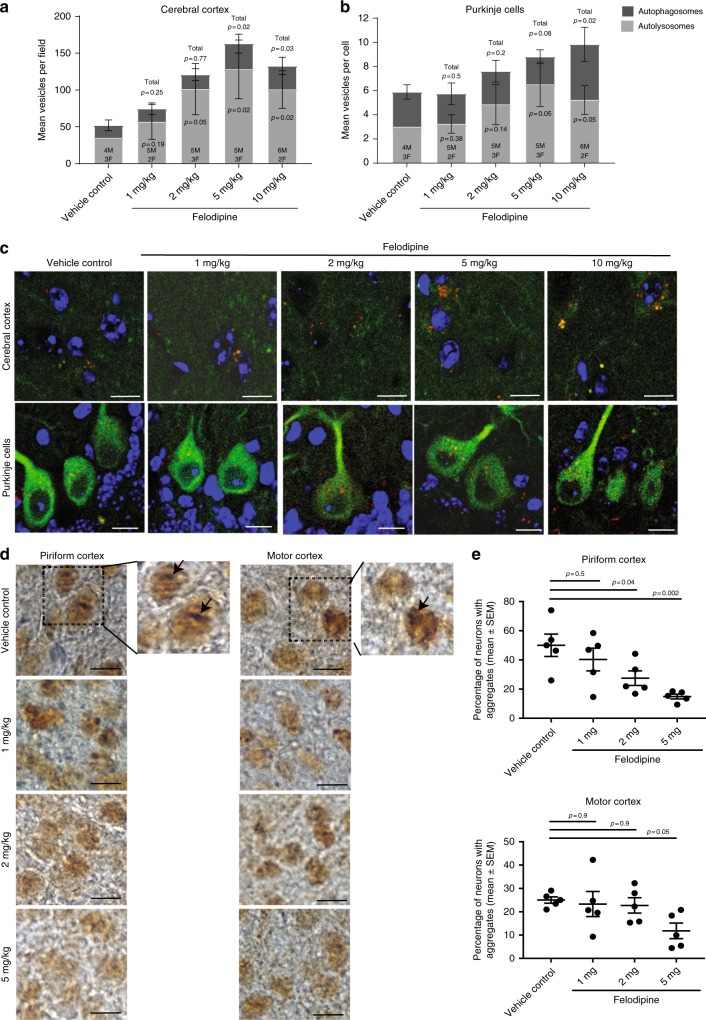
Felodipine in vivo dose-response experiments. **a**–**c** mRFP-GFP-LC3 transgenic mice (males and females, 6–7 weeks old) were injected i.p. with various doses of felodipine (1, 2, 5, 10 mg/kg body weight). **a**, **b** Treatment with 5 and 10 mg felodipine resulted in a statistically significant increase in autolysosomes and total vesicle number in the cerebral cortex with a similar trend observed in Purkinje cells. Autophagosome and autolysosome numbers are shown as mean +/− SEM; mean values of each vesicle type (autophagosome, autolysosome or total vesicles) for each dose of felodipine were compared with the mean value of same vesicle type to those in vehicle controls using one-sample, one-tailed unpaired *t*-test. Data shown was from 7 litters collected at different time-points (i.e., independent experiments), all values were normalised to mean autolysosome levels in the vehicle controls. **c** Representative images. Scale bar represents 10 µm in both cerebral cortex and Purkinje cells. **d**, **e** A six-week study was carried out on B6HD (N171–82Q) female mice. Mice were injected i.p. three times a week with 1, 2 or 5 mg/kg body weight of felodipine or vehicle control. Felodipine treatment resulted in a significant decrease in aggregates in the motor cortex (at 5 mg) and piriform cortex (2 and 5 mg). **d** Representative images of neuronal inclusions in piriform cortex and motor cortex of B6HD mice. Arrows indicate the neurons with huntingtin aggregates or inclusions, scale bar represents 10 µm. **e** Aggregate number was blind-counted in the motor cortex and piriform cortex. Data are presented as percentage of neurons with aggregates (mean +/− SEM) (*n* = 5 mice per group); analysed using one-way ANOVA with post hoc Dunnett’s multiple comparison test; exact *p* values for felodipine dose vs. control are shown

Having determined that felodipine effectively increased autophagic flux in primary neurons and rescued pathology in vivo via an autophagy-dependent mechanism, we went on to investigate the dose required in mice to induce autophagy and clear neurodegenerative-causing toxic proteins. Intraperitoneal administration of felodipine increased the numbers of autophagosomes and autolysosomes in mRFP-GFP-LC3 transgenic mouse brains 4 h after drug administration in a dose-dependent manner (Fig. 2a, b, c). Using the same dose range, we next performed a pilot study to determine whether and at what concentration felodipine was able to reduce the numbers of aggregates in Huntington’s disease transgenic mice. We used the N171-82Q mice (hereafter called B6HD mice), which express the first 171 amino acids of mutant huntingtin under the control of the mouse prion promoter, which restricts expression of the protein mainly to the brain^15^. The number of aggregates was significantly reduced in the piriform and motor cortex of these mice after 6 weeks treatment with intraperitoneal injections (Fig. 2d, e), paralleling what we have seen in other HD models when we induce autophagy^2^. These effects were dose-dependent, with the most effective dose determined to be 5 mg/kg. On the basis of these data, we continued to use 5 mg felodipine/kg body weight intraperitoneal injections (i.p.) for further studies. This dose of felodipine was effective at inducing autophagy at various ages (6 and 12 weeks old) in mRFP-GFP-LC3 mice analysed 4 h after injection and also in double transgenic mRFP-GFP-LC3/B6HD mice (Supplementary Fig. 3a, b, c) where these effects were also evident at 7.5 h after injection (Supplementary Fig. 3d).

### Felodipine efficacy study in N171-82Q (B6HD) mice

Since our data showed that i.p. injection of felodipine could reduce the levels of mutant huntingtin, the toxic causative agent in Huntington’s disease, we tested whether long-term treatment could ameliorate signs of disease in the B6HD Huntington’s disease mouse model^15^. These transgenic mice show a profound phenotype, including loss of motor coordination, including a marked decline in their ability to perform rotarod, grip-strength and wire-manoeuvre tasks, increased tremors, weight loss and premature death from about 12 weeks of age^16,17^. We pre-tested male mice at 5 weeks of age to estimate the baseline motor performances using rotarod testing, to ensure that the randomly assigned treatment and vehicle groups (also control wild-type littermates) were not significantly different in their ability to perform on the rotarod test. Felodipine treatment was then started at 6 weeks of age. The SHIRPA battery of behavioural testing including grip strength, wire manoeuvre and tremor monitoring was performed at 7, 9, 11, 13, 15, 17 and 19 weeks of age, while rotarod testing was performed every 4 weeks until death or euthanasia as required as a humane endpoint. All data of mice which were alive at these time-points were included in the analysis.

**Fig. 3:**
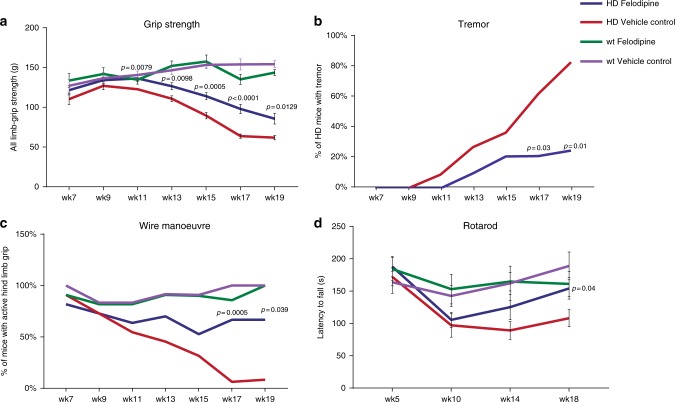
Efficacy study of felodipine in N171-82Q (B6HD) mice. B6HD male mice were given i.p. injections of 5 mg/kg felodipine or carrier substance (vehicle control) three times a week from 6 weeks of age. Wild-type mice littermates were also given felodipine or vehicle control injections with the same frequency. *n* = 22 mice for B6HD mice for both felodipine and vehicle control groups, while *n* = 11 for wild-type felodipine-treated littermates and *n* = 12 for wild-type vehicle-treated littermates. **a** Felodipine improved grip strength in B6HD mice. All B6HD mice showed some age dependent decline in grip strength compared to wild-type littermates regardless of treatment. However, felodipine significantly improved the decline in grip strength in B6HD mice relative to vehicle-treated B6HD mice at the time-points indicated. Data are shown as mean values +/− SEM; one-way ANOVA with post hoc Fisher’s LSD test; exact *p* values for B6HD-felodipine vs. B6HD-vehicle control are shown. Felodipine treatment had no effect on grip strength in wild-type mice. **b** The severity of tremors in B6HD mice was significantly improved by felodipine treatment at 17 and 19 weeks. Mann-Whitney two-tailed test was performed for ranked data; exact *p* values are shown. **c** Felodipine treatment improved performance at the wire manoeuvre task at 17 and 19 weeks in B6HD mice. Mice were scored on their ability to perform the wire manoeuvre task, data for the active grip with hind limbs are shown and analysed using one-way ANOVA for non-parametric data (Kruskal-Wallis with Dunn’s multiple comparison test); exact *p* values for felodipine vs. vehicle control in B6HD mice are shown. **d** Felodipine treatment improved rotarod performance in B6HD mice at 18 weeks. Data are presented as mean values +/− SEM; one-way ANOVA with post hoc Fisher’s LSD test; exact *p* values for felodipine vs. vehicle treated B6HD mice are shown. HD-vehicle control mice had a significantly reduced performance on the rotarod at week 10 and 14, compared to wild-type littermates (both felodipine-treated and vehicle-treated), as would be expected based on their known disease phenotypes

Felodipine-treated B6HD mice displayed significant improved in grip strength from 11 to 19 weeks of age (Fig. 3a, Supplementary Table 1). There was no influence of felodipine treatment on the performance of wild-type mice. The severity of tremors was significantly improved in felodipine-treated B6HD mice at 17 and 19 weeks of age and a trend towards an improvement was also seen at the age of 13 weeks (Fig. 3b, Supplementary Table 2). Wild-type mice do not demonstrate tremors at any age, also felodipine treatment did not induce tremors in the wild-type mice. The wire manoeuvre tests the muscle coordination and endurance of mice and has been adopted as part of the comprehensive SHIRPA phenotypic testing regimen^18^. It tests the capacity of mice to climb back on a horizontal wire when hung on the wire by their forelimbs. Felodipine-treated B6HD mice performed significantly better on the wire manoeuvre task at 17 and 19 weeks of age (Fig. 3c, Supplementary Table 3). There was no influence of felodipine treatment on the performance of wild-type mice. The accelerated rotarod apparatus is a measure of motor coordination and measures the ability of a mouse to maintain balance on a rotating cylinder (rotating towards the mouse). At 14 weeks of age, there was a trend towards an improved performance of the felodipine-treated B6HD animals. At 18 weeks of age, felodipine-treated B6HD mice stayed significantly longer on the rotarod than the vehicle-treated HD mice (Fig. 3d, Supplementary Table 4). Thus, felodipine improved the motor phenotype in B6HD mice in all the tests we have performed. Felodipine ameliorated the weight loss of the B6HD mice from 19 weeks but had no effects on weight of wild-type mice (Supplementary Fig. 4a, Supplementary Table 5). It was necessary to cull some mice by euthanasia at different time-points during the study due to adverse events e.g., due to swollen abdomen or abscesses after i.p. injections. Adverse events were observed in all groups irrespective of the genotype and drug treatment (Supplementary Table 6). This precluded meaningful assessment of effects on disease-associated life-span.

### Felodipine pharmacokinetics

**Fig. 4:**
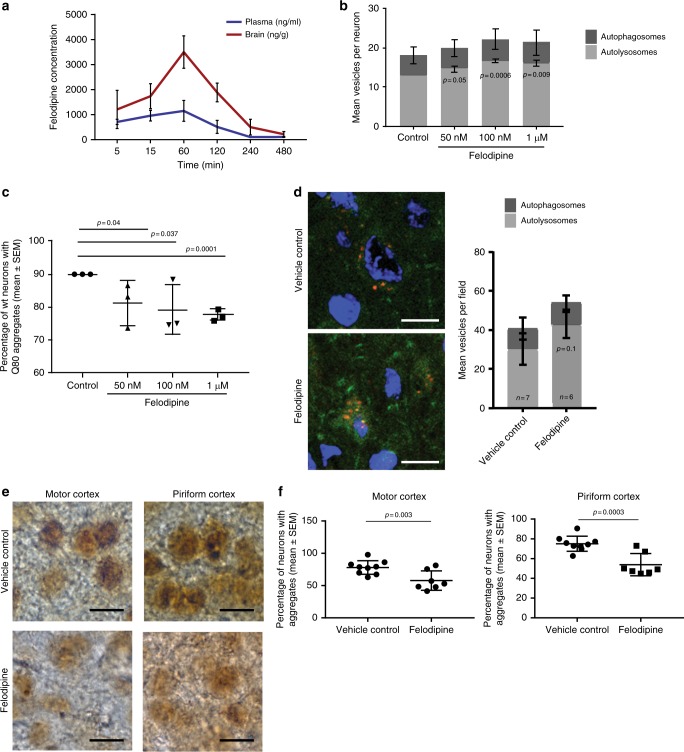
Felodipine pharmacokinetics and bioavailability. **a** Plasma and brain felodipine concentrations at 0, 5, 10, 60, 120, 240, and 480 min after single i.p. injection of 5 mg/kg body weight of felodipine in C57Bl6 wild-type mice, *n* = 3 mice per time-point. Means +/− SEM. **b** Felodipine (50 nM, 100 nM and 1 μM) in primary cortical neurons from mRFP-GFP-LC3 mice. Mean numbers of vesicles +/− SEM per neuron normalised to number of autolysosomes in DMSO (control); one sample, one-tailed unpaired *t*-test; exact *p* values for comparison of autolysosome numbers are shown. **c** Percentages of EGFP-positive neurons with aggregates (means +/− SEM (*n* = 3 independent experiments)). Data normalised to DMSO control in each experiment, set at 90. One-tailed unpaired *t*-test. **d**–**f** Double transgenic mRFP-GFP-LC3/B6HD male mice were implanted with felodipine-loaded minipumps (5 mg/kg/day) with 0.25 μl/h flow rate for 28 days. **d** Felodipine treatment increased the number of autolysosomes. Mean values +/− SEM; mean values for each vesicle type (autophagosomes, autolysosomes and total vesicles) for felodipine were compared with the same vesicle type of vehicle control, one-tailed unpaired *t*-test; exact *p* values are shown for autolysosomes. Only mice expressing detectable fluorescence of the mRFP-GFP-LC3 transgene were included in the final analysis. Representative images of cerebral cortex—scale bar = 10 µm. **e** Representative images of huntingtin inclusions in motor and piriform cortices. Scale bar = 10 µm. **f** Felodipine significantly reduced the percentage of cells with aggregates in both regions. Data are mean percentage of cells with aggregates per field with +/− SEM, using one-tailed unpaired *t*-test; exact *p* values for felodipine vs. vehicle control for each region are shown

Felodipine is an FDA-approved antihypertensive drug with a recommended dosing profile of 2.5 mg–20 mg/day^19^. We selected felodipine as the best candidate drug from a panel of L-type calcium channel blockers based on BBB-penetration and long half-life in man and therefore we predicted it would likely provide long-term upregulation of autophagy. However, we do not know how the pharmacokinetics in mice compare with those in man. In humans taking felodipine as an antihypertensive drug (10 mg daily dose), the peak plasma concentration in males is 28 nmol/L and is 33 nmol/L in females (average 30.5 nmol/L; approx. 11.7 ng/ml) in individuals aged 60–80 years-old^19,20^. However, how these relate to brain concentrations in humans is not clear from the literature and cannot be tested in human subjects. To better understand the correlation between plasma and brain concentration, we assessed the bioavailability of the drug in the mouse brain after 5 mg/kg body weight i.p. injection, using standard pharmacokinetic (PK) parameters. A *C*_max_ of 1151 ng/ml (2995.3 nmol/L) in plasma and 3500 ng/g (9108.4 nmol) in brain was achieved after 60 min of dosing via i.p. injection. The *T*_½_ was 0.9 h (54 min) in plasma and 1.8 h (1 h 48 min) in mouse brain. This gave an AUC^0-∞^ (area under the plasma concentration-time curve from time zero to extrapolated infinity) of 2540 ng/ml/h (6610 nmol/L/h) in plasma and 9330 ng/ml/h (24280.5 nmol/L/h) in brain (Table 2). We noticed that the felodipine brain concentration is almost double that of plasma after 15 min and is nearly three times higher in brain after 60 min (Table 3, Fig. 4a). The felodipine brain concentration 8 h after a single i.p. injection was 216.4 ± 107.6 ng/g (Table 3) and these pharmacokinetic parameters were sufficient to induce autophagic flux in mouse brain (Supplementary Fig. 3d shows autophagic flux increased 7.5 h after a single i.p. injection) and also to clear mutant huntingtin aggregates (Fig. 2d, e). Since it is not possible to test whether felodipine accumulates in the brain in man, we next performed similar studies using minipigs. This confirmed that felodipine accumulates in the brain, compared to the plasma (Supplementary Table 7, 8, and Supplementary Fig. 5a). This analysis found that more than 99% of the felodipine was protein bound in the plasma and the brain in mice.

**Table 2:** PK parameters of felodipine in mice

PK parameter	Units	Plasma	Brain
*C*_max_	ng/mL	1151	3500
*t*_max_	hours	1	1
*t*_1/2_	hours	0.9	1.8
T1	hours	1	1
T2	hours	4	8
AUC_0-t_	ng.h/ml	2406	8776
AUC_0-∞_	ng.h/ml	2540	9330

PK analysis was performed after single dose of 5 mg/kg body weight via i.p. injection. The PK parameters were calculated from the mean values from 3 mice at each time-point. Hence, these PK parameters have no SDs. SDs would not be appropriate, since the values at each time-point are from different mice. The spread of concentrations in the mice at each time-point is given in the analytical data (Table 3)

**Table 3:** Felodipine plasma and brain concentration in mice after single i.p. injection

Time (min)	Plasma (ng/ml)	Brain (ng/g)	Brain:plasma ratio
5	710.7 ± 105.0	1208.0 ± 760.8	2
15	957.7 ± 210.9	1738.3 ± 498.3	2
60	1151.0 ± 415.5	3500.0 ± 649.0	3
120	511.3 ± 260.9	1886.7 ± 373.5	4
240	105.5 ± 27.0	501.0 ± 314.6	5
480	106.7 ± 17.5	216.4 ± 107.6	2

Analysis was performed after single dose of 5 mg/kg body weight via i.p. injection (*n* = 3 mice per time-point). Data are presented as mean values ± SEM

These studies revealed that felodipine injected intraperitoneally in the mouse was rapidly metabolised but reached concentrations that are orders of magnitude higher than those seen in human. Therefore, we investigated whether we could induce autophagy in the mouse brain using concentrations of the drug with steady levels similar to those seen in humans. This was achieved by implanting drug-loaded subcutaneous Alzet osmotic minipumps. In all of these experiments, placebo-treated mice also had pumps implanted containing vehicle only and no drug. We measured the plasma and brain concentrations after 14 days (*n* = 4 mice) and also after 28 days (*n* = 8 mice) (Tables 4, 5). Both experiments further strengthened our previous observation that felodipine concentrates in the brain and its concentration is almost the double than that of plasma. Although there is large animal-to-animal variability in mice for the plasma and brain concentrations, we found that the average plasma concentration after 28 days was 12 ng/ml (31.22 nmol/L) which is very similar to the peak concentrations seen in humans i.e., approx. 11.7 ng/ml (30.5 nmol/L)^20^. In many mice, the concentrations were higher at day 7 than at 14 days (similarly at day 10 than at day 28). The mean brain concentration from both experiments with osmotic minipumps was approx. 42 ng/g (i.e., 109.3 nmol) in mice (Tables 4, 5).

**Table 4:** Felodipine plasma and brain concentration in mice during and after 14 day dosing via minipump

Mouse (*n*)	Plasma (ng/ml)		Brain (ng/g)
	Day 7	Day 14	Day 14
1	25.90	10.4	17.3
2	57.90	36.8	66
3	58.00	35.9	58.5
4	18.20	24.50	27.6
Mean	**40.00** ± **10.48**	**26.90** ± **6.17**	**42.35** ± **11.77**

Analysis was performed after 14 days of felodipine dosing of 5 mg/kg body weight/ day via osmotic minipump (Alzet Model 2002), flow rate 0.5 μl/h. Blood sample was collected at day 7 and day 14 (terminal). Data are presented as actual values for individual mice, and mean value +/− SEM shown in bold is provided in the final row. All osmotic minipumps in this study had no blockage, checked by assessing the remaining volume in the pump at day 14

**Table 5:** Felodipine plasma and brain concentration in mice during and after 28 days dosing via minipump

Mouse (*n*)	Plasma (ng/ml)		Brain (ng/g)
	Day 10	Day 28	Day 28
1	29.9	3.51	<40^a^
2	11.9	12.7	37.1
3	40.7	15.9	30.6
4	42.0	16.9	49.6
5	12.6	23.9	<40^a^
6	23.0	5.08	<40^a^
7	39.0	5.97	40.9
8	29.9	18.8	58.9
Mean	**28.625** ± **4.23**	**12.845** ± **2.59**	**43.42** ± **3.9**

Analysis was performed after 28 days of felodipine dosing of 5 mg/kg body weight/ day via osmotic minipump (Alzet Model 2002), flow rate 0.5 μl/h. Blood sample was collected at day 10 and day 28 (terminal) for analysis. Data is presented as actual values for individual mice and mean value +/− SEM shown in bold is provided in the final row. All osmotic minipumps in this study had no blockage, checked by remaining volume in the pump when replaced and at the end of the study

^a^indicates that samples which were below the limit of detection and were not included in the mean

### Felodipine increases autophagic flux at low concentrations

We were encouraged that the average plasma concentration in mice (31 nmol/L) mimicked the human peak plasma concentration and resulted in brain concentrations of approximately 109.3 nmol in mice implanted with the minipumps. Therefore, to test whether these concentrations were likely to be inducing autophagy, we examined the effects of low concentrations (50 and 100 nM) of felodipine in cultured primary neurons. Indeed, these concentrations increased the numbers of autolysosomes (suggesting increased autophagic flux) and significantly reduced the percentages of neurons with mutant huntingtin exon 1 aggregates (Fig. 4b, c). Then, we tested if the same was observed in vivo using osmotic minipump (Alzet Model 2004) administration of the drug in double transgenic mRFP-GFP-LC3/B6HD mice. When we restricted this cohort to those who had >5 ng/ml (13 nmol/L) plasma concentration (Supplementary Table 9), the mean number of autolysosomes was increased by felodipine, although this was not significant, likely due to the relatively low numbers of mice studied (Fig. 4d). This administration of felodipine using a minipump delivery strategy caused a significant decrease in the huntingtin aggregation in the B6HD mice, compared to that of vehicle-treated mice (Fig. 4e, f).

### Felodipine improves pathology of SNCA mice

**Fig. 5:**
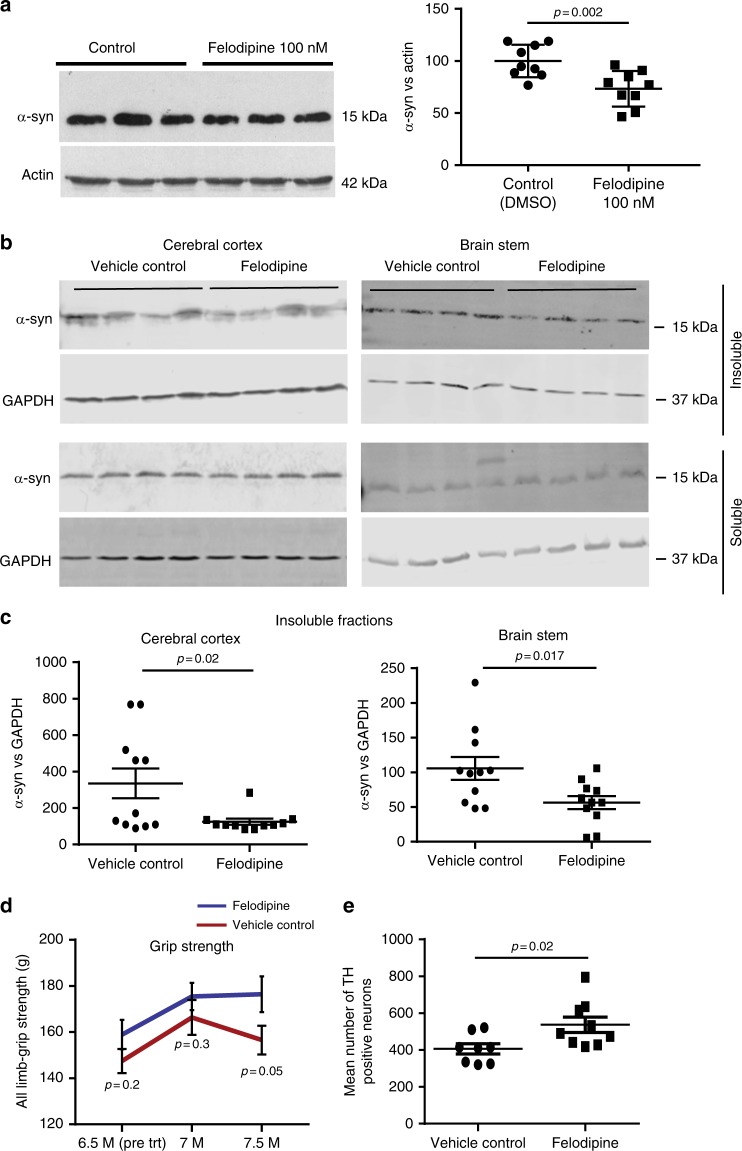
Felodipine clears mutant A53T a-synuclein in iPSC-derived human neurons and in vivo. **a** Representative western blots for α-synuclein in iPSC-derived human neurons homozygous for A53T α-synuclein mutation treated with 100 nM felodpine for 5 days. Densitometry data mean +/− SEM (α-synuclein vs. actin loading control; *n* = 3 independent experiments in triplicate; one-tailed, one sample *t*-test). Data normalised to control, set at 100. All nine data points are shown for the control and felodipine-treated samples. The data were also significant (*p* = 0.008) when each of the nine data points for control and treated samples were compared by *t* test. **b**–**e** Male and female SNCA mice (6.5 months-old; *n* = 20 felodipine; *n* = 19 vehicle) were implanted with subcutaneous felodipine-loaded osmotic minipumps (ALZET Model 2002) for 28 days, which were replaced once at day 14. At day 27, 17 felodipine and 18 vehicle control mice were available for analysis—some mice were culled for health reasons other than drug side effects. Behaviour was tested once before implanting and 14 days and 27 days after treatment (7 and 7.5 months-old, respectively). At the end of the study, 11 mice per group were used for biochemistry (**b**, **c**) and the remainder (9 felodipine; 8 vehicle) were used for histology. **b** Soluble and insoluble α-synuclein fractions of brain lysates from cerebral cortex and brain stem of SNCA mice. **c** Quantification of densitometry insoluble α-synuclein/GAPDH. Soluble α-synuclein was not significantly different between drug and vehicle (not shown). Data are mean α-synuclein vs. GAPDH ratio +/− SEM; two-tailed unpaired *t*-test. **d** Grip strength data are means +/− SEM; two-tailed, unpaired *t*-test. **e** Unbiased estimates of the number of TH-positive neurons in SN region of felodipine-treated and vehicle-treated SNCA mice. Data are means +/− SEM; two-tailed unpaired *t*-test. If the data are restricted to mice that survived until the end of the study, then the *p* values are: **c** (cerebral cortex): 0.057; (Brain stem): 0.025; **e**: 0.015

We aimed to test if this minipump dosing protocol that enables felodipine concentrations similar to those seen in humans would be able to ameliorate a disease caused by an autophagy substrate in a mouse model. Since drug administration had to be via minipump, this only gives us a window of 28 days to test the drug, as our Home Office licence (from the UK authority governing animal experimentation) does not permit replacing the pumps more than once for ethical reasons. Hence, we opted to study transgenic mice expressing the Parkinson’s disease-causing A53T mutant α-synuclein (α-syn) protein, an autophagy substrate^4^ (hereafter called SCNA mice), where pilot data had suggested that the disease shows progression over 28 days starting at 6.5 months. To confirm that low concentrations of felodipine (100 nM), similar to those which would be expected in the brains of humans taking this drug, we tested whether this concentration was sufficient to reduce α-synuclein levels in iPSC-derived human neurons that were engineered to be homozygous for the A53T α-synuclein mutation (Fig. 5a). Next, the SCNA mice were implanted with subcutaneous felodipine-loaded osmotic mini pumps (ALZET Model 2002) for 14 days, after which the pumps were replaced, and treatment was continued for another 14 days. This replacement protocol was chosen as it gives very similar concentrations to the single pumps used above but has a faster flow rate and more reliable drug administration. Felodipine decreased the level of insoluble fraction of α-syn vs. GAPDH in cerebral cortex and brain stem when compared to vehicle-treated mice (Fig. 5b, c). The SNCA mice in this study were relatively young (7.5 months-old; end time-point) and did not manifest obvious Lewy bodies with aggregates. These become apparent at later ages, for example 9 to 10 months^21^. The reduction in mutant A53T α-synuclein was accompanied by improved grip strength in these mice (Fig. 5d) and increased cell numbers in the substantia nigra (Fig. 5e).

## Discussion

Our data suggest that felodipine induces autophagy in neurons and enhances removal of a range of disease-causing proteins: mutant huntingtin, mutant α-synuclein and tau. Importantly our zebrafish data suggest that the rescue of tau toxicity is autophagy-dependent. A major attraction of drug repurposing is that this strategy offers the possibility of rapid transitioning from preclinical models to patients, as the toxicity and tolerability profiles of the relevant compounds are known. However, this approach assumes that the drug is working in its new indication via mechanisms and concentrations similar to its original/conventional application. We are not aware of any repurposing studies in the academic literature that have addressed this issue. Our data show that felodipine is more rapidly metabolised in mice (*T*_max_ 60 min, *T*_1/2_ 0.9 h) compared to in humans (*T*_max_ in 2.5–5 h, and *T*_1/2_ is 11–6 h), like many other drugs^22,23^. As a consequence, intraperitoneal administration results in transient exposures of the mice to the drug at concentrations that are more than two orders of magnitude higher than those observed in patients taking the drug for antihypertensive indications. This situation could result in effects of the drug in mice that would not be seen in humans taking conventional doses, for instance if the repurposing mechanism required drug concentrations that were much higher than those seen with standard dosing regimen. Such concentrations would also be likely associated with increased side-effect risks.

To circumvent this issue, we used subcutaneous minipumps to enable concentrations of the drug in the mouse plasma that were similar to those seen in the plasma of patients and at a steady state rather than wildly fluctuating concentrations.

We are not aware of any data on felodipine brain concentrations in humans. Accordingly, we have assessed brain/plasma concentrations of the compound in mice under i.p. and subcutaneous minipump regimes in mice and via oral dosing in minipigs and find that felodipine concentrates in the brain, hence we would speculate that this is likely to be the case in humans.

Our data with this minipump administration suggest that at human-like plasma concentrations, felodipine can induce autophagy in the brains of mice and clear aggregate-prone disease-causing proteins. Furthermore, felodipine ameliorates signs of disease and neurodegeneration in the A53T α-synuclein mouse model. As our UK Home Office licence for animal experimentation only permits a single replacement of the minipump, we were restricted to studying a model that had a disease course that had detectable decline and neurodegeneration in a one-month period and therefore precluded testing in models of HD or tauopathy. Felodipine is used as an antihypertensive drug, is administered long-term, and has relatively modest side-effect liabilities. Interestingly, epidemiological studies suggest that use of this drug may reduce the incidence of Parkinson’s disease in hypertensive patients^24^. While we are enthusiastic for it to be tested in people with neurodegenerative diseases, or individuals who are at risk of such conditions (e.g., presymptomatic carriers of the Huntington’s disease mutation), its side effects and dose will need to be considered, particularly in older patients where it is cleared more slowly. In conclusion, we believe that this is the first study showing that an approved drug at concentrations similar to those seen in human patients can induce autophagy in rodent brains, as well as the first study showing that an approved drug under such conditions can ameliorate neurodegenerative disease in a mouse model. These data support testing in humans with appropriate neurodegenerative diseases. Indeed, when a drug is safe and well tolerated, the possibility of preventive treatments aiming to delay disease onset also becomes an option.

## Methods

### Isolation and culture of mouse primary cortical neurons

Wild-type neuron cultures were generated from C57BL6 (Jackson Laboratories) embryos of E16.5 gestation. However, for primary mRFP-GFP-LC3 cortical neurons, transgenic mice were crossed with C57BL6 mice. At E16.5 gestation, females were sacrificed and embryos were harvested. Cortices from all embryos (regardless of genetic status) were combined to create mixed cultures^8^.

Briefly, brains were harvested and placed in Hank’s Balanced Salt Solution (HBSS), the meninges were removed and the cerebral cortices were dissected. After incubation in HBSS with 0.25% trypsin (Gibco) for 20 min at 37 °C, dissociated neurons were resuspended in HBSS and seeded on poly-D-lysine-coated 6-multiwell or 12-multiwell plates, or MatTek glass bottom culture dishes (P35G-1.0-14-C). Cells were cultured in maintenance media (Neurobasal-A medium (#12349015, Thermo Fisher Scientific) supplemented with 2 mM GlutaMAX, 200 mM B27 supplement and 1% Penicillin–Streptomycin) at 37 °C in a humidified incubator with 5% CO_2_. One half of the culture medium was changed every 2 days until drug treatment or lentivral infection. After 5 days of in vitro culture, differentiated neurons were either infected with lentiviral particles or treated with drugs as required by the experiment^25,26^.

### Imaging and quantification of mRFP-GFP-LC3 primary neuron

Primary neurons were treated either at DIV5 or DIV6 with 2X drug concentration in 0.5 ml fresh media, while keeping the half of the conditioned media (0.5 ml) in the well, in order to keep the final drug concentration correct. mRFP-GFP-LC3 neurons treated with the indicated concentration of the drugs were live-imaged after 2 h using confocal microscopy (63X NA 1.4 Plan Apochromat oil immersion lens; Carl Zeiss LSM710). At least 10 fields were imaged per treatment. Images were analysed by using Zen software. All saved images were coded by a different researcher and subsequently analysed blind. The number of yellow vesicles dots (GFP-positive and RFP-positive dots; autophagosomes) and red vesicles (RFP-only dots; autolysosomes) were counted in <50 neurons per treatment group. The mean number of autophagosomes and autolysosomes for each treatment group was calculated. Data was normalised to mean of the control autolysosome group to allow comparison between independent experiments. Statistical analysis was performed to compare the number of autolysosomes in treatment vs. control and total number of vesicles in treatment vs. control. A two-tailed *t*-test was used in the initial screen as it was not known whether or how the drugs would affect autophagy. Thereafter, one-tailed analysis was used in mRFP-GFP-LC3 vesicle counts since there was prior demonstration of autophagy upregulation.

### Transfection of wild-type primary neurons and quantification

After 5 days of in vitro culture, differentiated cortical neurons were infected with EGFP-Q80 lentiviral particles. Compounds were added 3 days after viral infection and left for a further 24 h. Neurons were fixed in a 2% PFA plus 7.5% glucose solution and coverslips were mounted with ProLong Gold antifade reagent with DAPI (Thermo Fisher; Invitrogen P36935). For each experiment, approximately 200 infected neurons for each treatment group were counted blind for the presence or absence of aggregates and the proportion of neurons with Q80 aggregates was determined. Data was normalised to mean of the control group to allow comparison between independent experiments. Statistical analysis was carried out using one-way ANOVA with Dunnett’s multiple comparison for the initial screen of L-type calcium channel blockers and one-tailed, unpaired t-test for subsequent felodipine treatment experiments.

### Human iPS cells

The human iPS WT line (KOLF-2) was generated by the Sanger Wellcome Institute Induced Pluripotent Stem Cell Initiative (HipsSci). Further information, including the Certificate of Analysis, can be found on the project website (www.hipsci.org). The A53T mutation in the human SNCA gene was generated by two base substitutions in this codon (GCA > ACC) using CRISPR/Cas9-induced homology directed repair in the kolf_2_C1 human iPSC line^27^. Cells were grown in TeSR-E8 (Stem Cell Technologies) on Synthemax (Corning) coated dishes, and expanded as clumps. Mutagenesis was achieved by nucleofection (Lonza, P3 kit, program CA137) of a single cell suspension of 106 cells with Cas9-crRNA-tracrRNA ribonucleoprotein (RNP) complexes. Synthetic RNA oligonucleotides (target site: 5′-GTGGTGCATGGTGTGGCAAC-3′, 225 pmol crRNA/tracrRNA, IDT) were annealed by heating to 95 °C for 2 min in duplex buffer (IDT) then cooled slowly, followed by addition of 122 pmol recombinant eSpCas9_1.1 protein (in 10 mM Tris-HCl, pH 7.4, 300 mM NaCl, 0.1 mM EDTA, 1 mM DTT), incubation at room temperature for 20 min, and addition of 500 pmol of a 100 nt ssDNA oligonucleotide (IDT Ultramer) as a homology-directed repair template to introduce the desired base change. After recovery, plating at single cell density and colony picking into 96 well plates, 288 clones were screened for heterozygous and homozygous mutations by high throughput sequencing of amplicons spanning the target site using an Illumina MiSeq instrument using primers A53TintF 5′-AGTTGTATTGAAAACTAGCTAATCAGC-3′ and A53TintR 5′-CATAGGAATCTTGAATACTGGGCC-3′. Final cell lines were expanded and further validated by a second round of sequencing. Independently targeted homozygous or heterozygous clones were used in downstream assays.

### Human iPS Cell culture and drug treatment

Human iPSCs were cultured in TeSR-E8 medium (Stem Cell Technologies) on Vitronectin (Thermo Fisher Scientific) coated plates. Cells were passaged with 0.5 mM EDTA when reaching 70% confluency at a ratio of 1:6. Differentiation into dopaminergic neurons was performed according to previously described protocols with slight modifications^28^. Briefly, iPSCs were dissociated into single cells and plated at 150,000 cells/cm^2^ on Matrigel (Thermo Fisher Scientific) coated plates. Cells were grown for 11 days in knockout serum replacement media (KSR) supplemented with LDN-193189 (100 nM, Stemgent), SB431542 (10 µM, R&D Systems), SAG (100 nM, Enzo Life Sciences), Purmorphamine (2 µM, Stemgent), FGF8a (100 ng/ml, R&D Systems) and CHIR99021 (3 µM, Stemgent) and 2 mM L-glutamine (Thermo Fisher Scientific). KSR medium was gradually changed to NNB medium containing Neurobasal medium, N2 (0.5×) and B27 (0.5×) from day 6. Media was changed to NB medium on day 12 containing Neurobasal medium, B27 (1×) and 2 mM L-glutamine (Thermo Fisher Scientific) supplemented with CHIR99021 (3 µM, Stemgent) (until day 14) and with BDNF (20 ng/ml, Peprotech), GDNF (20 ng/ml, Peprotech), Ascorbic Acid (200 µM, Sigma-Aldrich), TGFβ3, 1 ng/ml, Thermo Fisher Scientific), dibutyryl cAMP (500 µM; Sigma-Aldrich), and DAPT (10 µM, Stemgent). At day 21 cells were dissociated with StemPro Accutase (Thermo Fisher Scientific) and replated at 300,000 cells/cm2 in dishes pre-coated with Matrigel in final differentiation medium (NB with BDNF, GDNF, TGFb3, DAPT, dbcAMP and Ascorbic Acid). Cells were then cultured for 3 further weeks before treatments were initiated. Cells were treated with 100 nM felodipine and DMSO for 5 days. Drug was replenished after 48 h twice during the experiment. Cells (neurons) were collected and lysed in 2× Laemmmli buffer. Samples were analysed by western blotting with primary antibodies, rabbit anti-α-synuclein antibody (1:1000) (ab138501; Abcam) and mouse anti-actin antibody (1:5000) (ab3280, Abcam),

### Animal studies

All zebrafish and mouse procedures were performed in accordance with the UK Animals (Scientific Procedures) Act with appropriate Home Office Project and Personal animal licences and with local Ethics Committee approval. Zebrafish and mice were maintained and experiments were performed in accordance with ARRIVE guidelines. The protocols and procedures for the study of minipigs, were approved by the Animal Care and Use Committee, Guangzhou Institutes of Biomedicine and Health, Chinese Academy of Sciences.

*Maintenance of zebrafish stocks and transgenic lines:* Zebrafish were bred and maintained under standard conditions at 28.5 ± 0.5 °C on a 14 h light: 10 h dark cycle. Embryos were collected at 4–5 h post-fertilisation in embryo medium (EM) (5 mM NaCl, 0.17 mM KCl, 0.33 mMCaCl_2_, 0.33 mM Mg_2_SO_4_, 5 mM HEPES) and thereafter kept in a temperature controlled incubator at 28 °C and staged according to established criteria^29^. Generation of the Rho::EGFP-Tau^cu7^ was previously described^11^. The *atg7* (*atg7*^sa14768^) mutant fish line harbours a nonsense (C/T) mutation in exon 15 of 23 of *atg7*, resulting in a premature stop codon at amino acid 448 (of 697). Heterozygous fish were obtained from the Zebrafish Mutation Project^30^. This line was outcrossed for 2 generations, then crossed to Rho::EGFP-Tau^cu7^ fish to generate Rho::EGFP-Tau^cu7^;*atg7*^+/−^ fish. Fish carrying the *atg7* mutation were genotyped by allele-specific amplification using a custom-designed KASP assay from LGC Genomics. The UAS::Dendra-tauA152T^cu10^ line was generated as previously reported^13^. The pan-neuronal Gal4 driver line (hereafter referred to as PanN::Gal4VP16) was a kind gift from Herwig Baier (identified as line s1101tEt in the original publication^31^).

Outcrosses of Rho::EGFP-Tau^cu7^;*atg7*^+/−^ to *atg7*^+/−^ were used to generate wildtype or *atg7* knockout fish (*atg7*^+/+^ and *atg7*^−/−^, respectively) expressing EGFP-tau in the rod photoreceptors. Crosses of the responder fish UAS::Dendra-tauA152T with PanN::Gal4VP16 driver fish were used to generate experimental offspring with Dendra-tauA152T expressed throughout the central nervous system.

*Mice:* Mice were housed in individually ventilated cages with free access to standard animal food chow and water, in a climate-controlled room with a 12 h light/dark cycle. We used two different neurodegenerative disease mouse models and one mRFP-GFP-LC3 reporter line in this study. The autophagy reporter mRFP-GFP-LC3 mouse line was generated in our lab as previously described^8^. HD-N171–82Q mice, also called B6HD mice ((B6C3F1/J-Tg(HD82Gln)81 Dbo/J, Jackson Laboratory, Bar Harbour, ME, USA), carry an N-terminal fragment of the first 171 amino acids of human huntingtin with 82 glutamine repeats under the mouse prion promoter^15^. This line has been backcrossed on a C57BL/6J background for more than 10 generations. Previous work has shown that both male and female mice develop aggregates^17^ whereas male mice display more robust behavioural deficits^32^. Therefore, male mice were used for behavioural analysis and female mice were used for histological analysis. Hu α-syn (A53T) mice, also called SNCA(A53T)G2-3 line, or PrPsynA53T Tg mice (B6.Cg-Tg(Prnp-SNCA*A53T)23Mkle/J, from the Jackson Laboratory (Bar Harbour, ME, USA) express a 436 bp human α-synuclein cDNA bearing the familial Parkinson’s disease-linked A53T missense mutation driven by the mouse prion protein promoter^21^. SNCA mice were monitored daily to assess any adverse effects arising from drug treatment and to assess the symptoms of SNCA pathology. The following known symptoms of SNCA pathology were used as humane endpoints for the mice, which resulted in euthanasia: marked loss of appetite and fluid intake, staring coat, hunched posture, subdued behaviour, tremor, episodic seizures and dystonia. HD mouse endpoints are listed under B6HD mice efficacy trial below.

The litters of transgenic mice were genotyped from 3 weeks old onwards by PCR, according to the protocols recommended either by the Jackson Laboratory or by the authors the publications characterising these mice lines. The primer sequences for mRFP-GFP-LC3 mice genotyping are:

RFP-GFP-fwd: (5′-ACCTCCCACAACGAGGACTA-3′) and

RFP-GFP-rev: (5′-ACCTTGTGGCCGTTTACGTCG-3′).

*Minipigs:* Wild-type female Bama pigs, weighing 12–33 kg (Guangzhou Institutes of Biomedicine and Health, Chinese Academy of Sciences) were utilised for the studies. The protocol was approved by the Animal Care and Use Committee of GIBH. Animals were maintained on standard animal chow and water ad libitum, in a climate-controlled room (23 ± 1℃, 30–70% relative humidity, a minimum of 10 exchanges of room air per hour and a 12 h light/dark cycle) for one week prior to the experiments.

### Drug administration to animals

*Compound treatment in zebrafish transgenic lines:* Embryos from natural spawnings were collected in EM and reared at 28.5 °C. Larvae from the outcross of Rho::EGFP-Tau^cu7^;*atg7*^+/−^ to heterozygous *atg7*^+/−^ were treated from 1 d.p.f. with EM containing 0.03% phenylthiourea (PTU) to prevent pigmentation. At 3 d.p.f, larvae were screened for EGFP expression in the rod photoreceptors and then removed from PTU and reared in EM. Larvae were treated either with 10 µM verapamil, 0.1 µM felodipine or 0.1% DMSO from 4 to 10 d.p.f. Compounds and EM were refreshed daily. At 10 d.p.f., larvae were anaesthetised, and tails and heads were dissected. Heads were fixed in 4% PFA in PBS for the analysis of rod photoreceptor numbers. Tails were processed to extract genomic DNA and used for PCR analysis. Genotyping for *atg7* was performed by allele-specific amplification using reagents from a custom-designed KASP assay from LGC Genomics using previously described methods^30^. Dendra-tauA152T expressing embryos were reared in EM until 1 d.p.f. then treated with 0.1% DMSO, 10 µM verapamil or 0.1 µM felodipine either from 1 to 3 d.p.f. then processed for western blots and the quantification of phenotypes, or from 1 to 6 d.p.f. for analysis of sarkosyl soluble and insoluble fraction. Drugs and medium were replenished daily.

#### Felodipine administration to mice and minipigs

##### via i.p. injection

Felodipine (Sigma F9677) for i.p. injections was prepared as a stock solution of 20 mg/ml in 20% ethanol. On the injection day, this was diluted to 1 or 0.5 mg/ml in 0.15 M NaCl, 5% Tween-20 and 5% PEG400 freshly before injection.

##### via subcutaneous osmotic minipump

Felodipine (Sigma F9677) solution in 49% DMSO and 49% PEG300, 2% Tween20 was given subcutaneously as a continuous infusion with a daily dose of 5 mg/kg body weight by implanting osmotic pumps (0.25 or 0.5 μl/h flow rate, Alzet Model 2004 and 2002, respectively). Felodipine concentration in the minipump was adjusted according to each animal’s body weight to obtain a delivery of 5 mg/kg body weight /day and the pumps were primed by soaking in saline at 37 °C overnight.

##### via gastric gavage to minipigs

Felodipine was dissolved in a solution containing 2% DMSO, 4% ethanol, 4% castor oil and 90% ddH_2_O. Animals were randomly distributed into 4 experimental groups (*n* = 2). All groups were given 2.5 mg/kg orally by gastric gavage.

### B6HD mice efficacy trial

Treated and untreated mice, as well as wild-type and transgenic littermates were housed together and identified by ear notches. Thus, observer was blind to their treatment and genetic status during behavioural testing. 22 B6HD male transgenic mice were used for felodipine treatment and 22 transgenic male animals were used for the control (vehicle) group. We also included 11 wild-type male mice (littermates) for felodipine and 12 vehicle (control) treated male mice to study the effect of felodipine on behavioural parameters in non-diseased animals. At 5 weeks of age, a pre-assessment of motor performances using rotarod was performed. There were no significant differences in test performances in the mice assigned to the treatment and placebo groups at 5 weeks. Felodipine treatment was started at 6 weeks age. The mice were weighed and received i.p. injections (5 mg/kg body weight felodipine) three times a week (Monday, Wednesday and Friday). The vehicle control group received i.p. injections with the carrier substance (0.15 M NaCl, 5% Tween-20 and 5% PEG 400 and 2% ethanol). Mice were monitored daily to assess any adverse effects arising from drug treatment and to assess the symptoms of HD pathology. The following known symptoms of HD pathology were used as humane endpoints for the mice, which resulted in euthanasia: marked loss of appetite and fluid intake, staring coat, hunched posture, tremor, subdued behaviour, or 15% weight loss over a period of less than 3 days (see Supplementary Table 6).

### Behavioural testing

We assessed rotarod motor performance at week 10, 14 and 18, with one pre-trial testing at week 5 (Accelerating Model, Ugo Basile, Biological Research Apparatus, Varese, Italy), as described in^17^. Tests for grip strength, wire manoeuvre and tremor monitoring were performed at 7, 9, 11, 13, 15, 17, 19 and 21 weeks of age. Wire manoeuvre and tremor are part of the SHIRPA battery of behavioural tests^18^. Grip strength was monitored quantitatively by using a grip strength metre (Biosep, France), as described^17^. Mice were scored on their ability to perform the wire manoeuvre task, scores were set as: 0, active grip with hind legs; 1, difficulty grasping with hind legs; 2, unable to lift hind legs; 3, falls within 30 s; 4, falls immediately.

### Blood and brain sampling of mice and minipigs

*Mice:* 10 week old wild-type male mice (C57Bl/6J) were i.p. injected with 5 mg/ kg body weight felodipine then anaesthetised using isoflurane at 6 different time-points (i.e., 5 min, 15 min, 60 min, 120 min, 240 min, and 8 h). Once anaesthetised, a terminal blood sample was taken by cardiac puncture. Blood (0.3–0.8 ml) was collected in EDTA tubes (Microvette 100K3E, Sarstedt), The plasma fraction was immediately separated by centrifugation (3000 rpm, 5 min, 4 °C), placed on dry ice then stored at −80 °C until LC-MS analysis to determine drug concentration. After confirming death by cervical dislocation, the brains were collected and frozen on dry ice then stored at −80 °C until LC-MS analysis.

Repeat blood sampling (via saphenous vein) was performed during the course of continuous dosing of felodipine via osmotic minipump according to the experiment (either at day 10 and 20 for Alzet Model 2002 or day 14 for Alzet Model 2004) followed by collection of a terminal sample at day 28. On each occasion, blood samples of 100–120 μl was collected in EDTA tubes (Microvette 300K2E, Sarstedt). The plasma fraction was separated and stored as described above.

Pharmacokinetic analysis for PK parameters of plasma and brain was performed by Q3 Analytical Ltd.

*Minipigs:* After administration of felodipine, whole blood samples (500 μl) were obtained from the orbital venous plexus for terminal sampling at the following time-points after dosing: 2, 4, 6 and 8 h and from the jugular vein for serial collection at 5, 15, 30 min and 1, 2, 4, 6, 8, 12 and 24 h from the same animal. Whole blood samples were collected in heparinized tubes. The plasma fraction was immediately separated by centrifugation (3000 rpm, 6 min, 4 °C) and stored at −20 °C until LC-MS analysis. The animals were culled humanely by euthanasia by carbon dioxide 24 h after experiment.

### Analysis of zebrafish experiments

*Imaging and quantification of photoreceptors of zebrafish:* 10 μm transverse cryosections through the zebrafish retina were cut using a Bright cryostat. Sections were examined to identify the optic nerve head (central retina) and 5 sections at the level of the optic nerve head were imaged using a Zeiss Axioplan2 fluorescent microscope equipped with a QImaging Retiga 2000 R digital camera. Fluorescence images of the GFP-signal were then analysed by Fiji software (ImageJ) with binary options and automatic quantification in specific regions of interest (ROIs) corresponding to the photoreceptor layer.

*Tau sarkosyl extraction for zebrafish:* Tau soluble and insoluble fractionation was performed using the sarkosyl extraction protocol^33^ with minor modifications as described in Lopez et al.^13^. Samples were run on 10% acrylamide SDS-PAGE gels. Blots were blocked in 5% non-fat milk in PBS-T and incubated with primary antibodies; mouse antibody Tau5 (1:1000) (ab80579, Abcam) and mouse anti-tubulin (1:2000) (T9026, Sigma-Aldrich).

### Analysis of brains from mouse experiments

For immunohistochemistry and fluorescent imaging of autophagosomes and autolysosomes, mice were perfused with ice-cold PBS and 4% paraformaldehyde/PBS (w/v, pH 7.4) after deep anaesthesia by i.p. injection of pentobarbital sodium (euthatal). Brains were removed and post fixed for 1–2 h in the same fixative. After cryoprotection in 30% sucrose/PBS (w/v, pH 7.4) for 2–3 days at 4 °C, brains were frozen on dry ice and stored in −80 °C. For analysis of soluble and insoluble α-synuclein, the mice were humanely killed by a schedule 1 method and brains were collected.

### Immunohistochemistry and quantification of mouse brains

For mutant huntingtin aggregate analysis, 30 μm thick coronal sections of cortex of HD mice brain (felodipine-and vehicle-treated) were analysed for neuronal inclusions according to established protocols^17,34^. Sections were labelled with anti-huntingtin antibody by free-floating immunohistochemistry (EM48, Chemicon). Staining was performed by peroxidase labelling using Vectastain Avidin:Biotinylated enzyme complex (ABC) kit and visualised with DAB reagent (Vector Laboratories). Mutant huntingtin inclusions were counted blind in the piriform cortex and motor cortex in three fields on five sections per animal at a magnification of X100 (Zeiss Axioskop2, field diameter 0.2 mm). The percentage of cells with aggregates was normalised to the mean percentage of vehicle control to allow comparison between independent experiments. Statistical analysis was carried out using one-way ANOVA with Dunnett’s multiple comparison in initial experiments (Fig. 2d, e) and by one-tailed, unpaired, *t*-test thereafter (Fig. 4e, f).

For TH IHC labelling of the substantia nigra of SNCA mice brains, serial coronal sections (50 μm thick, bregma −2.46 to −4.04) were cut using a cryostat. Every sixth section was selected and antigen retrieval was performed by heating to 100 °C in 1X sodium citrate buffer, pH 6.0 in PBS (Sigma) for 20 min. In order to inactivate the endogenous peroxidase activity, the sections were incubated in 3% hydrogen peroxide in 20% methanol for 20 min at room temperature. Sections were blocked with 5% goat serum (Sigma-Aldrich)/PBS plus 0.1% Triton X-100 (v/v) for 30 min, followed by avidin/biotin blocking, as instructed by manufacturer (Vector Laboratories). The sections were washed 3 times with 1X PBS after each step above, where each wash was for 5–10 min. Finally, the sections were incubated with TH antibody (ab112; Abcam) 1:200 in 5% goat serum in PBS plus 0.1% Triton X-100 (v/v) overnight, followed by incubation with biotin-conjugated secondary antibodies to rabbit, ABC reagents (Vector Laboratories), and visualised with DAB reagent (Vector Laboratories). Free floating stained sections were then mounted on gelatin-coated slides, air dried and then counterstained with Nissl (0.09% thionin, w/v) as described previously^35^. Sections were dehydrated in 100% ethanol and cleared in Xylene (Fisher Scientific) followed by mounting with DPX (BDH).

Unbiased estimates of the number of TH-positive neurons from the SN region were determined using optical dissector method. A counting frame of 150 μm × 150 μm was placed in a systematic random fashion throughout SN regions (both left and right side simultaneously). This method was carried out at ×20 magnification using a microscope imaging system (Olympus BX53) equipped with a computer-controlled motorised stage and Stereo Investigator software (MicroBrightField). The total count of TH-stained neurons in substantia nigra was quantified and mean values were calculated for all the sections for each mouse. Data was analysed by two-tailed unpaired *t*-test.

### Quantification of mRFP-GFP-LC3 vesicles in mouse brains

For analysis of autophagosomes and autolysosomes in the brains of mRFP-GFP-LC3 mice, 8 μm thick sagittal sections were cut from perfused, cryo-protected mRFP-GFP-LC3 mouse brains. Sections were mounted on poly-lysine coated slides and air dried for 2 h at room temperature. To reduce auto fluorescence, sections were stained in 0.05% Sudan Black in 70% methanol for 5–10 min^36^. Sections were air dried for 10 min and mounted using Citifluor AF1 with DAPI (1:1000). Sections were imaged using a confocal LSM 710 (Carl Zeiss), equipped with a digital camera and operated with ZEN imaging software. All slides were imaged using the same settings, except for minor changes on Digital Offset, if needed.

Five confocal images for cerebral cortex and 4–5 images for Purkinje cells (covering 20 neurons) were quantified. The number of mRFP-GFP-LC3 dots (autophagosomes, yellow) and mRFP-LC3 dots (autolysosomes, red) were counted manually within a single frame for cerebral cortex and in various fames covering 20 Purkinje neurons, switching between the red and green channels to confirm that each yellow vesicle is also red (otherwise ignoring the yellow vesicles which were not red) while counting. Numbers of autophagosomes were quantified per frame for cortex and per neuron for Purkinje cells, as the latter are easily discriminated. ZEN 2011 imaging software was used for counting. The experimenter was blind to the treatments or slide names or numbers. To avoid inter-operator variability within an experiment, only one person counted all the replicates of one experiment.

In some cases, samples were collected from different litters over a short period of time; therefore analysis was performed as a single experiment (Supplementary Fig. 3a, b and d). Alternatively, when samples were collected over an extended period of time, values were normalised to the mean of autolysosome levels of the vehicle control of that individual experimental group (as in Fig. 2a, b, c, Fig. 4d and Supplementary Fig. 3c). Mean values of each vesicle type (autophagosomes, autolysosomes and total vesicles) of felodipine-treated mice were compared to the mean value of the same vesicle type of vehicle-treated mice (control). Statistical analysis was performed comparing autolysosome number or total vesicle number for treatment vs. control using one sample one-tailed unpaired *t*-test, since we had prior evidence that felodipine treatment would increase autophagy.

When we analysed double transgenic mRFP-GFP-LC3/B6HD mice, we analysed only males implanted with felodipine-loaded minipumps (5 mg/kg/day) with 0.25 μl/h flow rate for 28 days. One hemisphere was analysed for mRFP-GFP-LC3 vesicles, while the other was analysed for huntingtin aggregates.

### α-synuclein soluble and insoluble extraction

SNCA (A53T) G2–3 mice brain regions (cerebral cortex and brain stem) were dissected out and soluble and insoluble fractionation was performed using the modified protocol as described^37^. Protein concentration was determined using the Bradford assay and 40 μg of protein was loaded onto 15% acrylamide SDS-PAGE gels. The same vehicle-treated control mouse sample was loaded on each gel to allow normalisation between independent blots. Blots were blocked in 5% non-fat milk in PBS-T and incubated with primary antibodies, rabbit anti-α-synuclein antibody (1:1000) (ab138501; Abcam); mouse anti-GAPDH antibody (1:5000) (ab8245, Abcam), followed by appropriate fluorescent secondary antibodies and analysed with a LICOR-Odyssey apparatus using IMAGE STUDIO Lite software, which enables quantitative analysis of blotting signals. GAPDH was used as loading control for both fractions.

### Statistical analysis

Data are presented as mean values ± SEM (standard error mean) or SD (standard deviation) calculated using Microscoft Excel and GraphPad Prism 7 software. *p* < 0.05 was considered as the threshold for statistical significance. Exact *p* values are provided throughout. Statistical analysis was performed using one tailed or two-tailed Student’s *t*-test or one-way ANOVA followed by appropriate post hoc test for multiple comparisons using GraphPad Prism 7 unless otherwise stated.

### Reporting summary

Further information on experimental design is available in the Nature Research Reporting Summary linked to this article.

## Supplementary information

Supplementary Information

Peer Review File

Reporting Summary

Source Data

## Data Availability

The data that support the findings of this study are available from the corresponding author upon reasonable request.
