# Author Correction: Felodipine induces autophagy in mouse brains with pharmacokinetics amenable to repurposing

**DOI:** 10.1038/s41467-019-10536-y

**Published:** 2019-06-04

**Authors:** Farah H. Siddiqi, Fiona M. Menzies, Ana Lopez, Eleanna Stamatakou, Cansu Karabiyik, Rodrigo Ureshino, Thomas Ricketts, Maria Jimenez-Sanchez, Miguel Angel Esteban, Liangxue Lai, Micky D. Tortorella, Zhiwei Luo, Hao Liu, Emmanouil Metzakopian, Hugo J. R. Fernandes, Andrew Bassett, Eric Karran, Bruce L. Miller, Angeleen Fleming, David C. Rubinsztein

**Affiliations:** 1 Department of Medical Genetics, Cambridge Institute for Medical Research, University of Cambridge, The Keith Peters Building, Cambridge Biomedical Campus, Hills Road, Cambridge, CB2 0XY UK; 2 UK Dementia Research Institute, Cambridge Institute for Medical Research, University of Cambridge, The Keith Peters Building, Cambridge Biomedical Campus, Hills Road, Cambridge, CB2 0XY UK; 3 Department of Physiology, Development and Neuroscience, University of Cambridge, Downing Street, Cambridge, CB2 3DY UK; 4 Guangzhou Institutes of Biomedicine and Health, Chinese Academy of Sciences, 190 Kai Yuan Avenue, Science Park, 501530 Guangzhou, China; 5 UK Dementia Research Institute, Department of Clinical Neurosciences, University of Cambridge, Cambridge, CB2 0AH UK; 6 Wellcome Sanger Institute, Wellcome Genome Campus, Hinxton, Cambridgeshire CB10 1SA UK; 7 AbbVie Inc., Foundational Neuroscience Center, 200 Sidney Street, Cambridge, MA 02139 USA; 8 Memory and Aging Center, Department of Neurology, University of California, San Francisco, CA USA; 9 Present Address: Department of Basic and Clinical Neuroscience, King’s College London, Institute of Psychiatry, Psychology and Neuroscience, Maurice Wohl Clinical Neuroscience Institute, London, SE5 9RX UK

**Keywords:** Pharmacology, Pharmacology, Target validation, Target validation, Neurological disorders

Correction to: *Nature Communications* 10.1038/s41467-019-09494-2, Published online 18 April 2019.

The original version of this Article omitted the following from the Acknowledgements:

The John Black Charitable Foundation and The Robert Luff Foundation.

This has now been corrected in both the PDF and HTML versions of the Article.

