# Supplementary material for: Felodipine induces autophagy in mouse brains with pharmacokinetics amenable to repurposing
Source: Nat Commun. 2019 Apr 18;10:1817. doi: 10.1038/s41467-019-09494-2 (PMC6472390; doi:10.1038/s41467-019-09494-2)
Supplement: Supplementary file 1 — Supplementary Information [file 41467_2019_9494_MOESM1_ESM.pdf]

# Supplementary Materials

# Felodipine induces autophagy in mouse brains with pharmacokinetics amenable to repurposing

Farah H Siddiqi[1,2], Fiona M. Menzies[1], Ana Lopez[1,3], Eleanna Stamatakou[1,2], Cansu Karabiyik[1], Rodrigo Ureshino[1,3], Thomas Ricketts[1], Maria Jimenez-Sanchez[1,4], Miguel Angel Esteban[5], Liangxue Lai[5], Micky D Tortorella[5], Zhiwei Luo[5], Hao Liu[5], Emmanouil Metzakopian[6], Hugo J R Fernandes[6], Andrew Bassett[7], Eric Karran[8], Bruce L. Miller[9], Angeleen Fleming[1,3] and David C. Rubinsztein[1,2]*

## Supplementary Figure 1

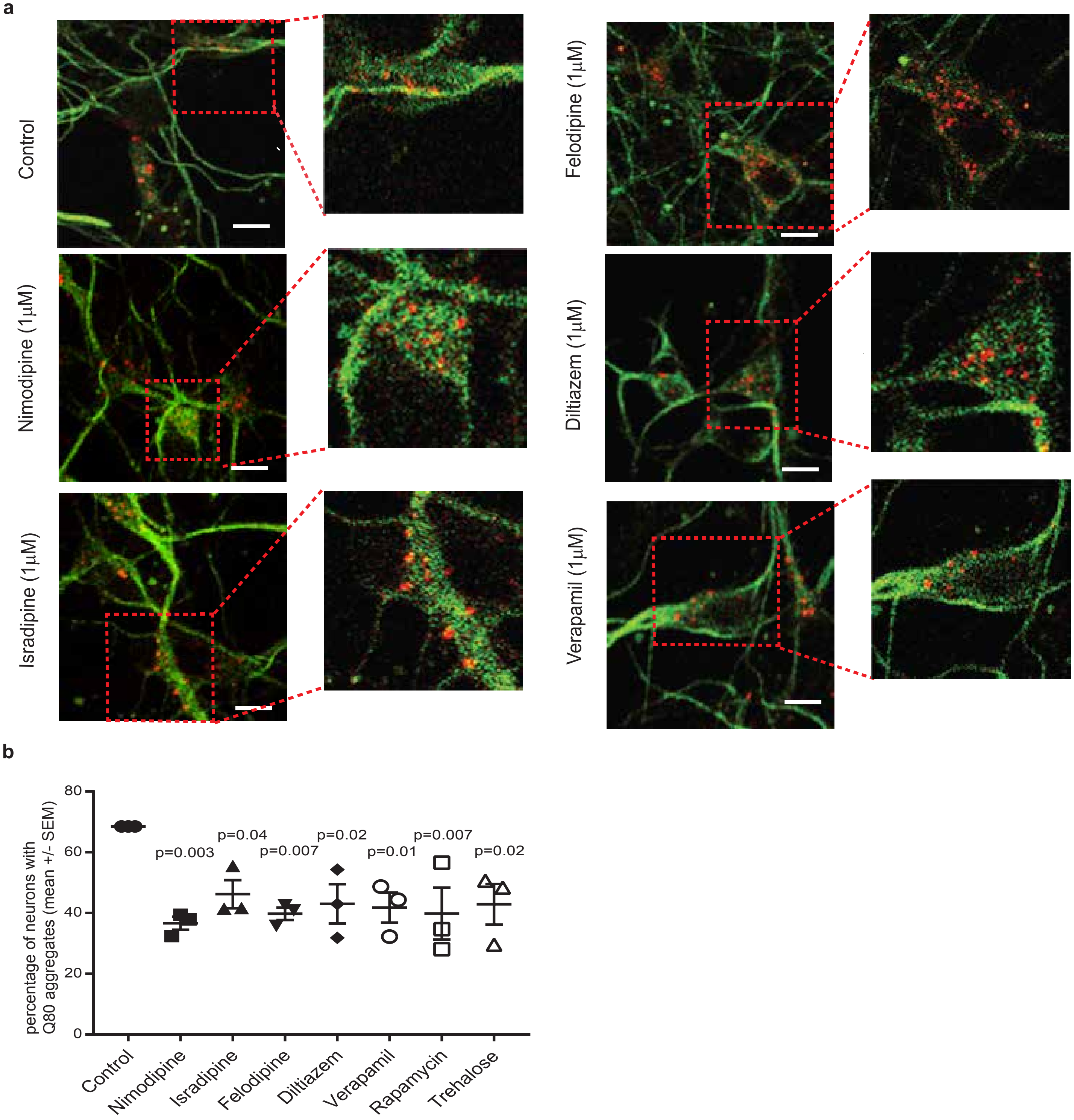


**Supplementary Figure 1: Screening of L-type calcium channel blockers in primary neurons.**

**a** Representative confocal images of live cortical primary neurons from mRFP-GFP-LC3 transgenic mice treated with a panel of different L-type calcium channel blockers showing autolysosomes (red-only vesicles) and autophagosomes (yellow vesicles) indicated by red and yellow arrows, respectively. Scale bar represents 10 µm.

**b** Primary cortical neurons (from wild type mice) were infected with EGFP-Q80 lentivirus. After 72 hrs of infection, neurons were treated with a panel of calcium channel blockers (at 1 µM) or DMSO (control) for 24 hrs. All the drugs tested produced a significant decrease in the number cells containing aggregates. The percentage of EGFP-positive cells with aggregates are shown as mean +/- SEM (n=3 independent experiments); mean values for compounds were compared with the mean of control, using one-way ANOVA with post hoc Dunnett's multiple comparison test), where $p< 0.05$ was considered significant. Exact p values for drug vs control are shown.

## Supplementary Figure 2

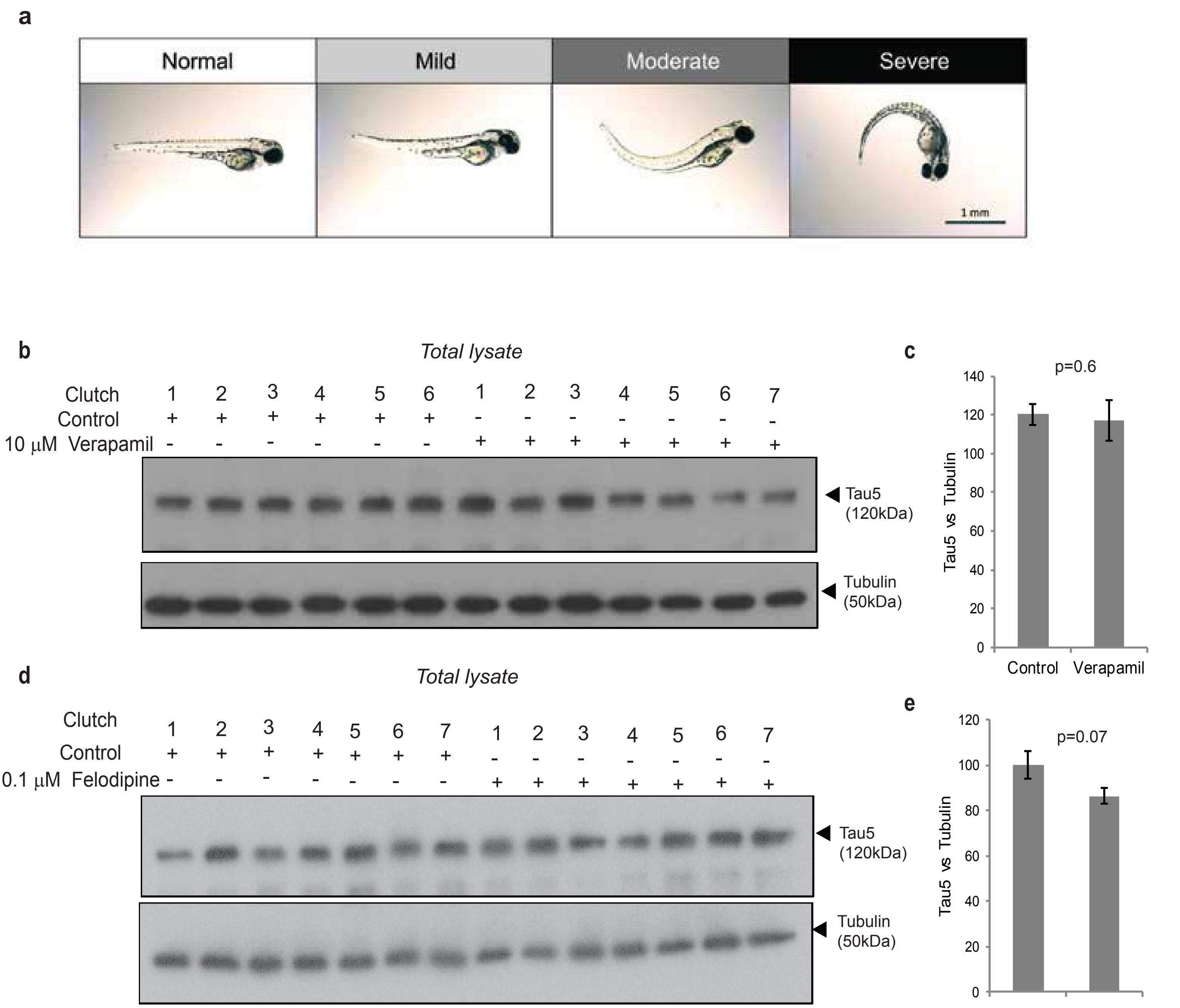


**Supplementary Figure 2: Analysis of L-type calcium channel blockers in primary neurons and zebrafish**

**a** Representative images of different phenotypes found in fish expressing dendra-A152T-tau and used to score morphological defects upon different treatments (see ref. 13 for detailed description of phenotypes). Scale bar represents 1mm.

**b and c** Levels of total tau from Dendra-tauA152T expressing zebrafish were slightly decreased (not significant) after 10 µM verapamil treatment compared to DMSO control. **b** Western blots for Tau5 to detect tau level in 6 d.p.f. fish from 6 independent clutches (10 fish/group) treated either with DMSO or 10 µM verapamil. Tubulin was used as loading control. **c** Densitometry of total tau vs tubulin of the western blot shown in **b**.

**d and e** Levels of total tau of Dendra-tauA152T protein were slightly decreased (not significant) after 0.1 µM felodipine treatment compared to DMSO control. D) Western blots for Tau5 to detect tau level in 6 d.p.f. fish from 7 independent clutches (10 fish/group) treated either with DMSO or 0.1 µM felodipine. Tubulin was used as loading control. **e** Densitometry of total tau vs tubulin of the western blot shown in **d**.

## Supplementary Figure 3

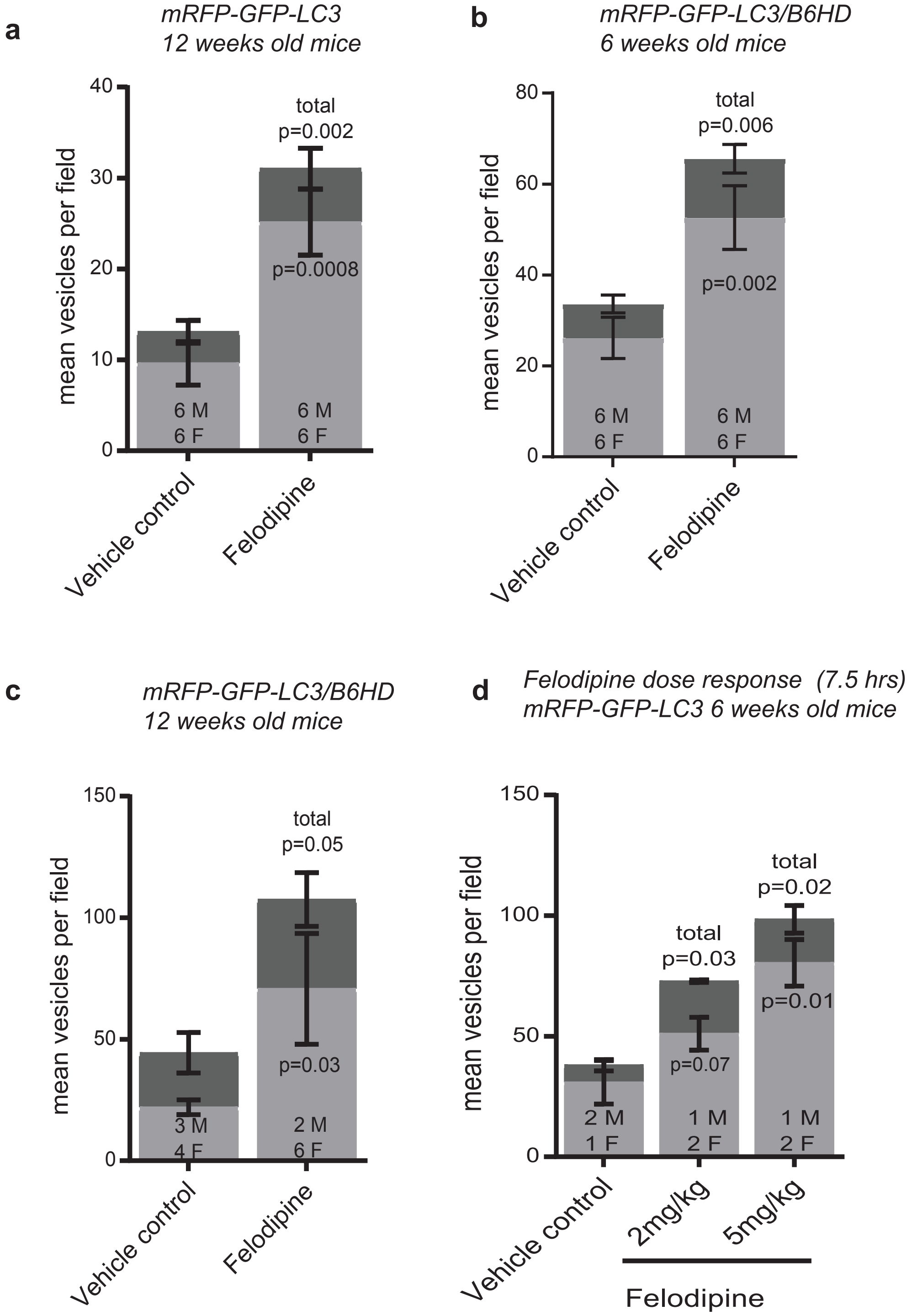


**Supplementary Figure 3: Felodipine increases autophagy in cerebral cortex in mice**

mRFP-GFP-LC3 or mRFP-GFP-LC3/B6HD double transgenic mice (males and females) were injected i.p. with felodipine (5 mg/kg in **a, b**, and **c** ; or doses as indicated in **d**) or vehicle control. A to C analysis was performed 4 hours after i.p. injection, while in D, 6-7 weeks old mRFP-GFP-LC3 mice were analysed 7.5 hrs after injection. Felodipine treatment increased the number of autolysosomes and the total number of vesicles in all experiments. Autophagosome and autolysosome numbers are shown as mean values +/- SEM. Mean values of each vesicle type (autophagosomes, autolysosomes and total vesicles) of felodipine-treated mice were compared to the mean value of the same vesicle type of vehicle-treated mice (control), using one-tailed unpaired *t*-test; exact p values for autolysosome and total vesicle comparisons are shown. All values were normalised to mean autolysosome levels in the vehicle controls. Samples in **a, b** and **d** were analysed as a single experiment. In **c**, samples were collected from individual litters over a long period of time therefore values were normalised to the mean of autolysosome level of the vehicle control of each litter.

# Supplementary Figure 4

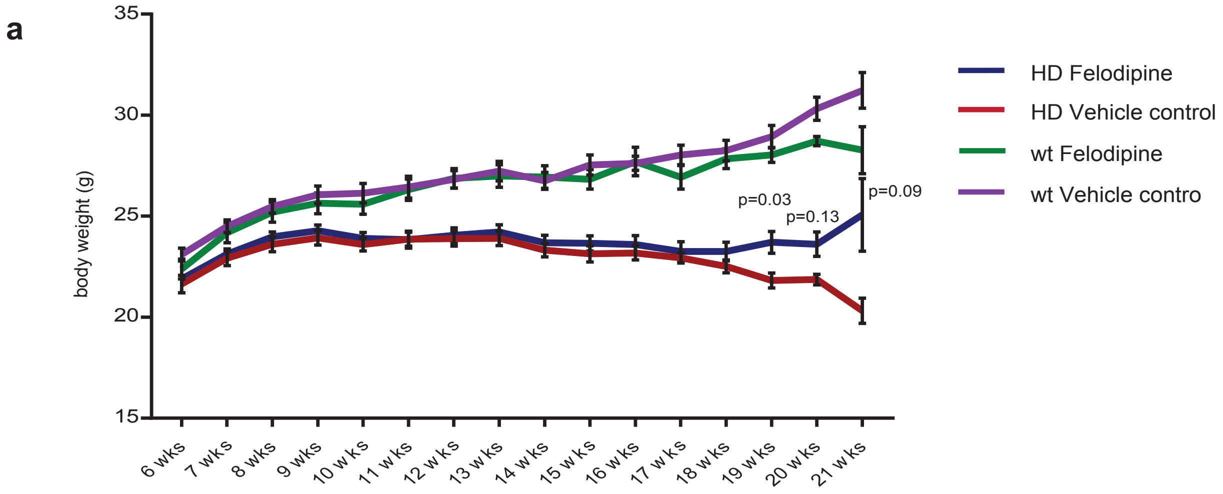


**Supplementary Figure 4: Felodipine efficacy trial in N171-82Q (B6HD) mice.**

**a** Body weight data over the period of efficacy trial. Data presented as mean values +/- SEM; one-tailed, unpaired *t*-test for felodipine-B6HD mice vs vehicle-B6HD mice; exact p values are shown. At week 20 and 21, the p values are non-significant due to increased mortality from various reasons (not correlated with genotype or treatment, See Supplementary Table 6), resulting reduction in number of mice per group.

## Supplementary Figure 5

**a**

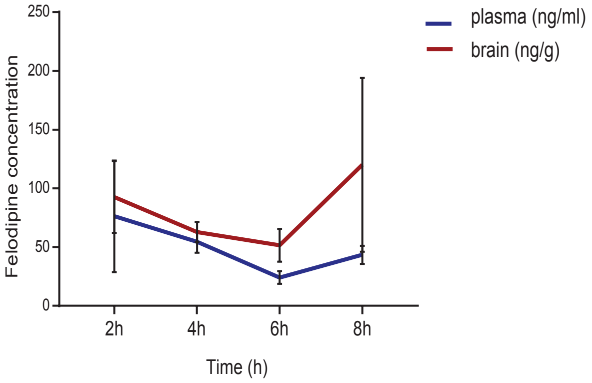


**Supplementary Figure 5: Felodipine pharmacokinetics**

**a** Plasma concentration in minipigs, dosed at 2.5 mg/kg body weight orally. Data presented as mean values +/- SD. Blood was collected serially at 2, 4, 6 and 8 hrs after dosing. n=2 animals per time point.

# Supplementary Figure 6   Full scans of uncropped blots

## Fig 1b

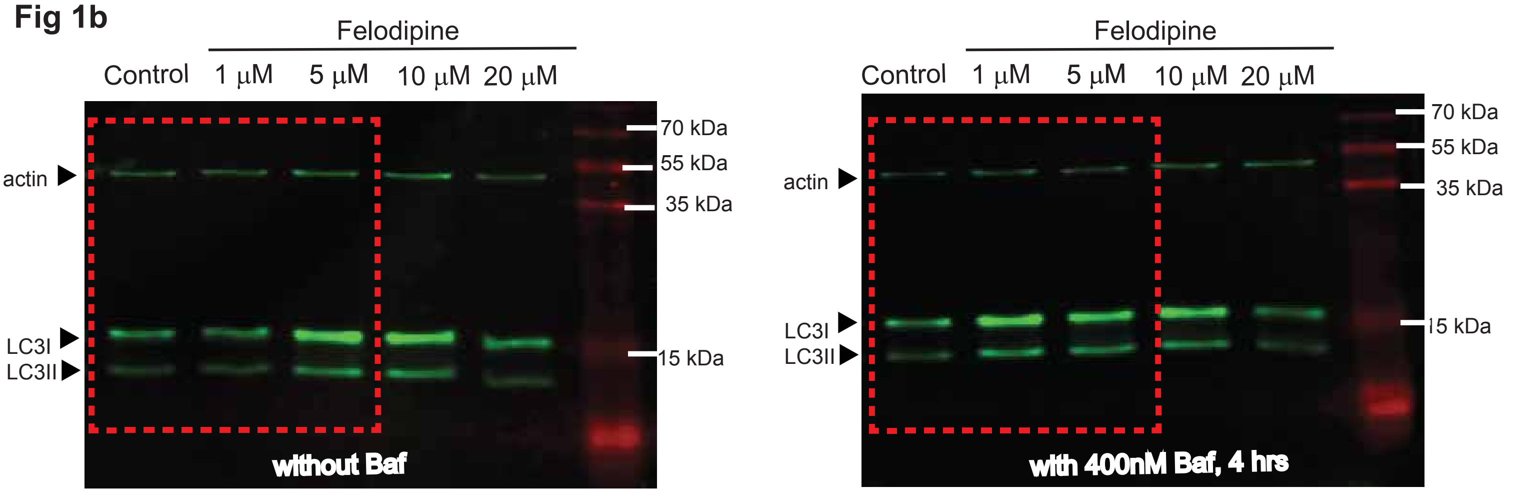


## Fig 1g

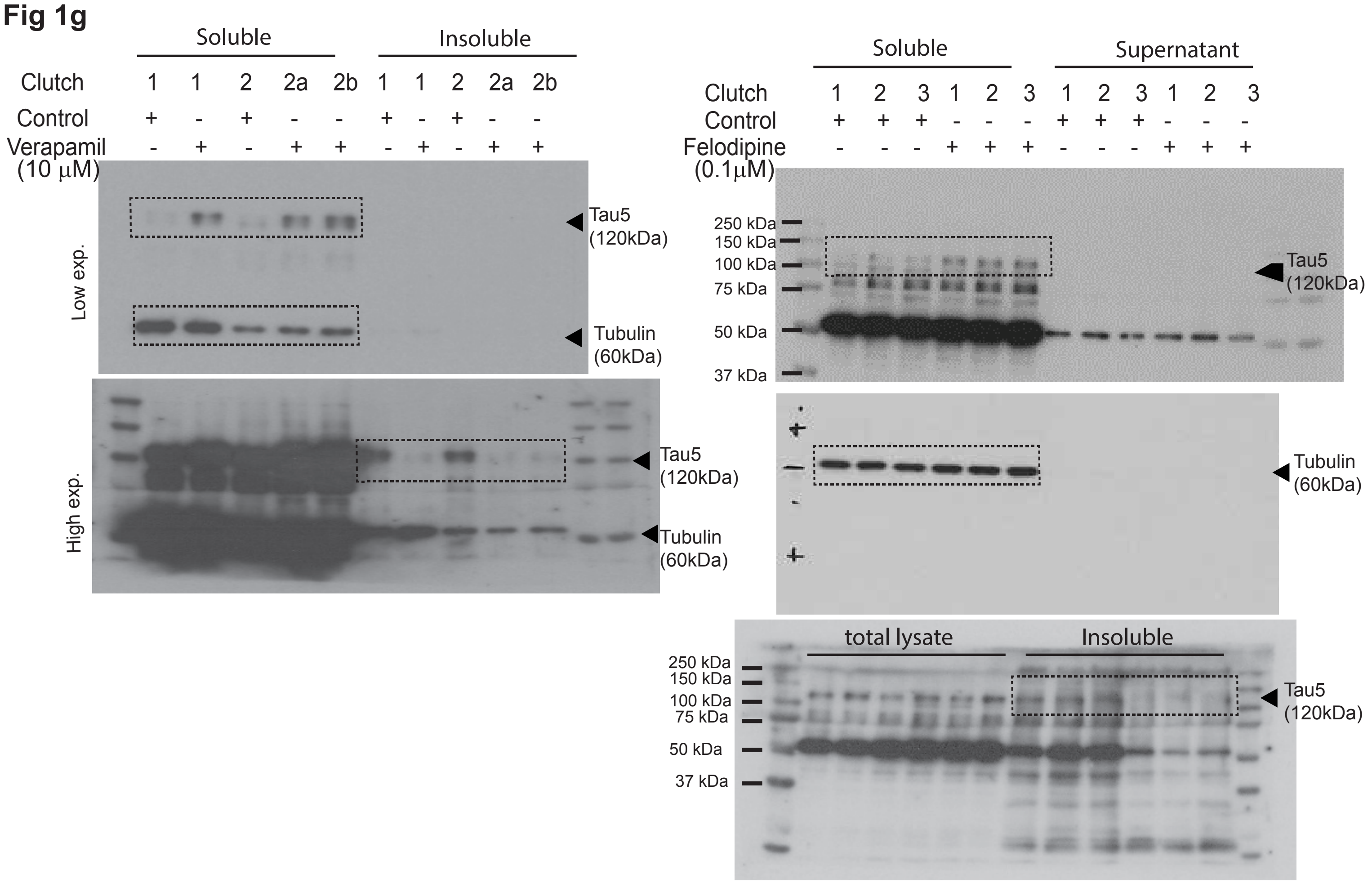

## Supplementary Figure 6_continued Full scans of uncropped blots

**Fig 5a**

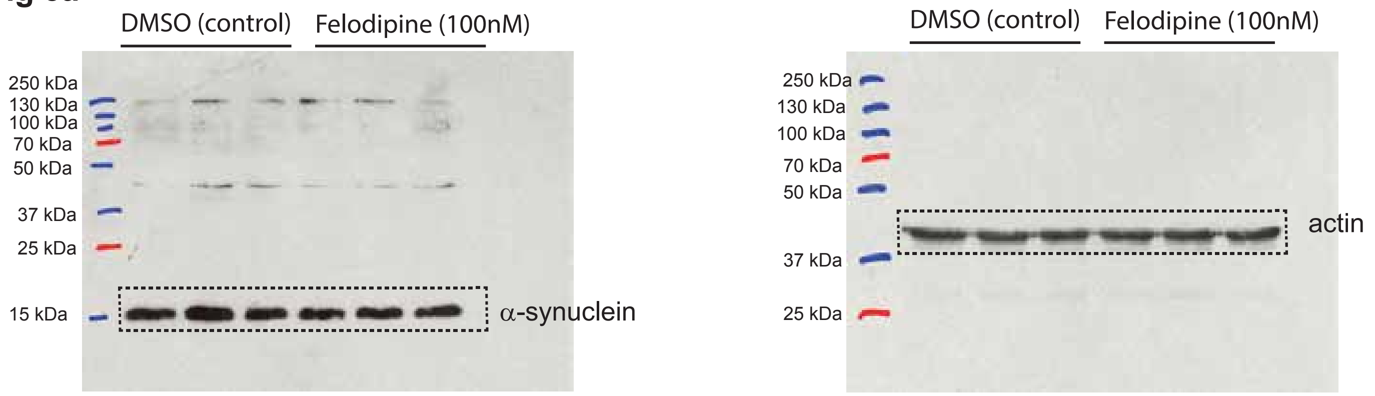


**Fig 5b**

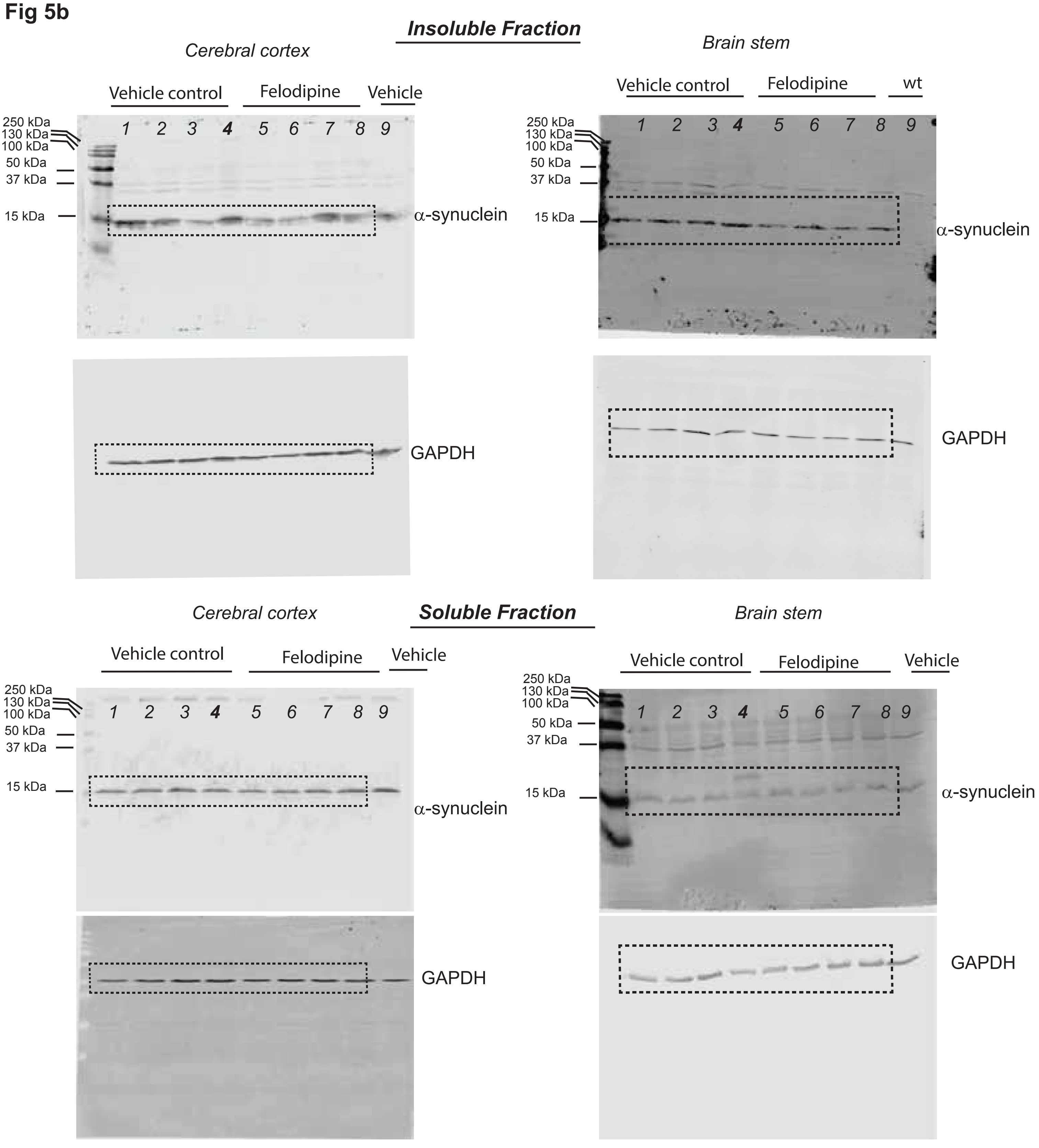

## Supplementary Figure 6_continued   Full scans of uncropped blots

### Supplementory Fig 2b

*Total lysate*

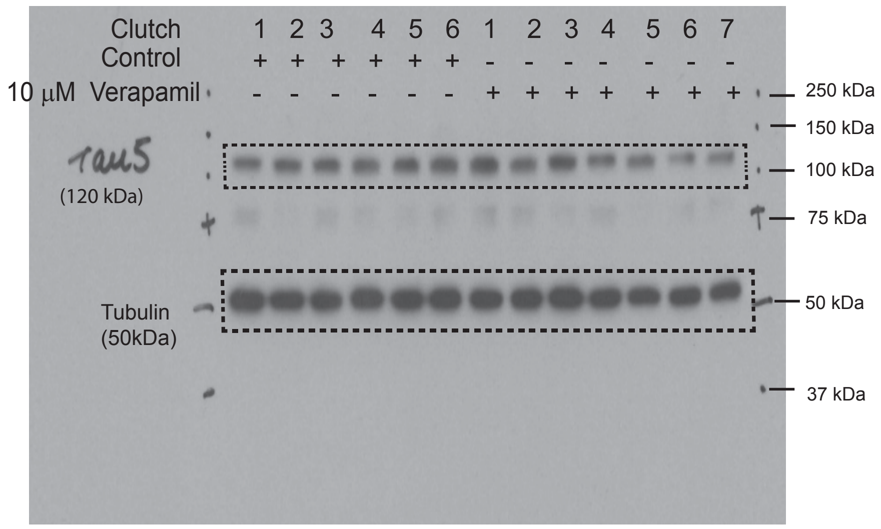


### Supplementory Fig 2d

*Total lysate*

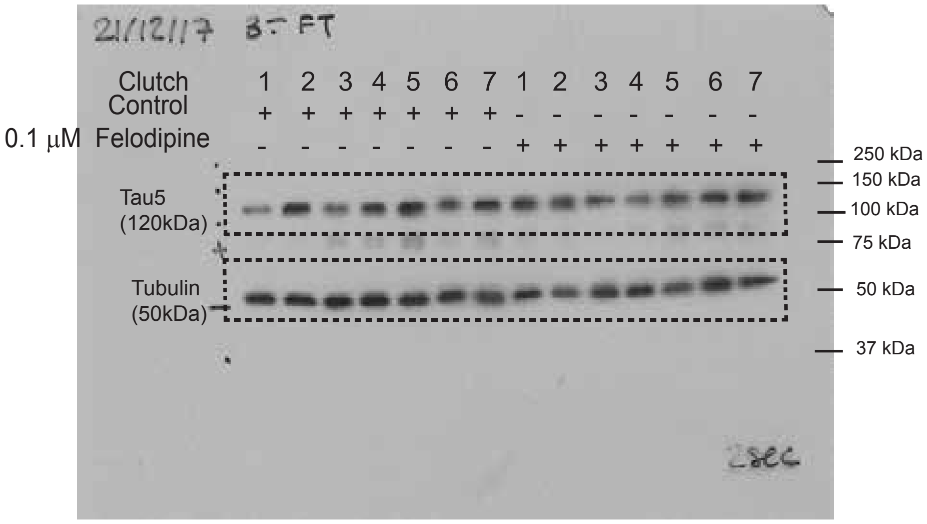

**Supplementary Table 1: p values showing multiple comparison of grip strength analysis of B6HD mice after i.p. injections in felodipine efficacy study**

| All Limbs | p values | | | | | | |
|---|---|---|---|---|---|---|---|
| **One-way ANOVA (Fisher's LSD test)** | **wk 7** | **wk 9** | **wk 11** | **wk 13** | **wk 15** | **wk 17** | **wk 19** |
| HD Felodipine vs. HD Vehicle control | 0.2054 | 0.3032 | 0.0079 | 0.0098 | 0.0005 | <0.0001 | 0.0129 |
| HD Felodipine vs. wt Felodipine | 0.2642 | 0.3592 | 0.7740 | 0.0007 | <0.0001 | 0.0005 | <0.0001 |
| HD Felodipine vs. wt Vehicle control | 0.6007 | 0.7715 | 0.4785 | 0.0056 | <0.0001 | <0.0001 | <0.0001 |
| HD Vehicle control vs. wt Felodipine | 0.0337 | 0.0814 | 0.0523 | <0.0001 | <0.0001 | <0.0001 | <0.0001 |
| HD Vehicle control vs. wt Vehicle control | 0.1143 | 0.2489 | 0.0033 | <0.0001 | <0.0001 | <0.0001 | <0.0001 |
| wt Felodipine vs. wt Vehicle control | 0.5881 | 0.5732 | 0.3848 | 0.4974 | 0.6386 | 0.0857 | 0.4431 |

**Supplementary Table 2: p values showing multiple comparison of tremor analysis of B6HD mice after i.p. injections in felodipine efficacy study**

| Tremor | p values | | | | | | |
|---|---|---|---|---|---|---|---|
| **Mann Whitney test (two-tailed test)** | **wk 7** | **wk 9** | **wk 11** | **wk 13** | **wk 15** | **wk 17** | **wk 19** |
| HD Felodipine vs. HD Vehicle control | >0.9999 | >0.9999 | >0.9999 | 0.2379 | 0.4756 | 0.0329 | 0.0123 |

**Supplementary Table 3: p values showing multiple comparison of wire manoeuvre analysis of B6HD mice after i.p. injections in felodipine efficacy study**

| **Wire manoeuvre** | **p values** | | | | | | |
|---|---|---|---|---|---|---|---|
| **ANOVA non-parametric (Kruskal-Wallis, Dunn's multiple comparisons test)** | **wk 7** | **wk 9** | **wk 11** | **wk 13** | **wk 15** | **wk 17** | **wk 19** |
| HD Felodipine vs. HD Vehicle control | >0.9999 | >0.9999 | >0.9999 | 0.7287 | >0.9999 | 0.0005 | 0.0393 |
| HD Felodipine vs. wt Felodipine | >0.9999 | >0.9999 | >0.9999 | >0.9999 | 0.4896 | >0.9999 | >0.9999 |
| HD Felodipine vs. wt Vehicle control | 0.5802 | >0.9999 | >0.9999 | 0.9715 | 0.3787 | >0.9999 | >0.9999 |
| HD Vehicle control vs. wt Felodipine | >0.9999 | >0.9999 | 0.7750 | 0.0783 | 0.0338 | 0.0023 | 0.0151 |
| HD Vehicle control vs. wt Vehicle control | >0.9999 | >0.9999 | 0.4207 | 0.0351 | 0.0193 | <0.0001 | 0.0015 |
| wt Felodipine vs. wt Vehicle control | >0.9999 | >0.9999 | >0.9999 | >0.9999 | >0.9999 | >0.9999 | >0.9999 |

**Supplementary Table 4: p values showing multiple comparison of rotarod analysis of B6HD mice after i.p. injections in felodipine efficacy study**

| Rotarod | p values | | | |
|---|---|---|---|---|
| **One-way ANOVA (Fisher's LSD test)** | **wk5** | **wk10** | **wk14** | **wk18** |
| HD Felodipine vs. HD Vehicle control | 0.4504 | 0.7223 | 0.1371 | 0.0445 |
| HD Felodipine vs. wt Felodipine | 0.8784 | 0.0939 | 0.1605 | 0.7848 |
| HD Felodipine vs. wt Vehicle control | 0.3269 | 0.1875 | 0.1886 | 0.1813 |
| HD Vehicle control vs. wt Felodipine | 0.5923 | 0.0361 | 0.0058 | 0.0328 |
| HD Vehicle control vs. wt Vehicle control | 0.7187 | 0.0847 | 0.0075 | 0.0018 |
| wt Felodipine vs. wt Vehicle control | 0.4335 | 0.7229 | 0.9303 | 0.3113 |

**Supplementary Table 5: p values showing multiple comparison of body weight B6HD mice after i.p. injections in felodipine efficacy study**

| **Body weight (g)** | **p values** | | | | | | | | | | | | | | | |
|---|---|---|---|---|---|---|---|---|---|---|---|---|---|---|---|---|
| **One-way ANOVA (Fisher's LSD test)** | **wk6** | **wk7** | **wk8** | **wk9** | **wk10** | **wk11** | **wk12** | **wk13** | **wk14** | **wk15** | **wk16** | **wk17** | **wk`8** | **wk19** | **wk20** | **wk21** |
| HD Felodipine vs. HD Vehicle control | 0.6092 | 0.6415 | 0.4051 | 0.4526 | 0.5035 | 0.9656 | 0.7445 | 0.5339 | 0.4673 | 0.3608 | 0.5250 | 0.6134 | 0.2772 | 0.0324 | 0.1259 | 0.0857 |
| HD Felodipine vs. wt Felodipine | 0.4011 | 0.0542 | 0.0263 | 0.0194 | 0.0036 | 0.0004 | <0.0001 | <0.0001 | <0.0001 | <0.0001 | <0.0001 | <0.0001 | <0.0001 | 0.0002 | 0.0073 | 0.2585 |
| HD Felodipine vs. wt Vehicle control | 0.0376 | 0.0104 | 0.0053 | 0.0019 | 0.0001 | 0.0002 | <0.0001 | <0.0001 | <0.0001 | <0.0001 | <0.0001 | <0.0001 | <0.0001 | <0.0001 | <0.0001 | 0.0292 |
| HD Vehicle control vs. wt Felodipine | 0.2106 | 0.0223 | 0.0043 | 0.0037 | 0.0007 | 0.0005 | <0.0001 | <0.0001 | <0.0001 | <0.0001 | <0.0001 | <0.0001 | <0.0001 | <0.0001 | 0.0009 | 0.0123 |
| HD Vehicle control vs. wt Vehicle control | 0.0130 | 0.0035 | 0.0006 | 0.0003 | <0.0001 | 0.0002 | <0.0001 | <0.0001 | <0.0001 | <0.0001 | <0.0001 | <0.0001 | <0.0001 | <0.0001 | <0.0001 | 0.0009 |
| wt Felodipine vs. wt Vehicle control | 0.2851 | 0.5949 | 0.6412 | 0.5080 | 0.3892 | 0.8737 | 0.9426 | 0.7379 | 0.7861 | 0.3644 | 0.9178 | 0.1949 | 0.6627 | 0.4029 | 0.3897 | 0.2304 |

## Supplementary Table 6: Humane endpoints in B6HD mice after i.p. injections in felodipine efficacy study

| genotype | drug | no. of mice (n) | % of mice euthanised due to HD symptoms (1) | average and range of age (wks) of mice euthanised due to HD symptoms | % of mice euthanised due to multiple i.p. injection (2) | average and range of age (wks) of mice euthanised due to multiple i.p. injection | % of mice euthanised due to other reasons (3) | average and range of age (wks) euthanised due to other reasons | % of mice euthanised at the end of study | average age (weeks) euthanised at the end of study |
|---|---|---|---|---|---|---|---|---|---|---|
| HD | Felodpine | 22 | 71.4 | 18.5 (14.7-23.7 ) | 9.5 | 19.8 (19.5- 20.0) | 19 | 13.8 (10.7-19.0) | 0.0 | 0.0 |
| HD | Vehicle control | 22 | 95.2 | 18.0 ( 14.3-21.1) | 0.0 | 0.0 | 4.7 | 20.0 (20.0-20.0) | 0.0 | 0.0 |
| wt | Felodipine | 11 | 0.0 | 0.0 | 18.2 | 18.3 (18.3-18.3) | 72.7 | 17.4 (14.3- 20) | 9.1 | 23.7 (23.7- 23.7) |
| wt | Vehicle control | 12 | 0.0 | 0.0 | 25 | 18.4 (14.5-20.5) | 58.3 | 18.1 (14.1-20.6) | 16.6 | 23.5 (23.4 23.7) |

(1) tremor, subdued, hunched, pilo are classical HD symptoms.
(2) stiff and swollen abdomen were considered as side effect of multiple i.p. injection.
(3) other causes include diarrhoea, prolapsed penis, eye infection, swollen snout, perpetual abscess, swollen anus

**Supplementary Table 7: PK parameters of felodipine in minipigs**

| PK parameters of felodipine in minipigs. | | |
|---|---|---|
| **PK Parameter** | **Units** | **Plasma** |
| $C_{max}$ | ng/mL | 275.9 ± 121.13 |
| $t_{max}$ | hours | 0.938 ± 0.38 |
| $t_{1/2}$ | hours | 7.751 ± 2.21 |
| T1 | hours | 1 |
| T2 | hours | 24 |
| $AUC_{0-t}$ | ng.hr/ml | 973.221 ± 370.87 |
| $AUC_{0-\infty}$ | ng.hr/ml | 1006.132 ± 384.85 |

**Supplementary Table 7:** PK parameters of felodipine in minipigs. PK analysis was performed after single dose of 2.5 mg/kg body weight orally (n= 4 minipigs per time point).  Table provides mean values +/-SEM of PK profile of same animals at each time point.

**Supplementary Table 8: Felodipine brain and plasma concentration in minipigs.**

| Time (hrs) | plasma (ng/ml) | brain (ng/g) | Brain: Plasma Ratio |
|---|---|---|---|
| 2 | 76.23 ± 67.22 | 92.6 ± 42.99 | 1.21 |
| 4 | 54.52 ± 13.24 | 62.75 ± 12.23 | 1.15 |
| 6 | 24.08 ± 7.57 | 51.55 ± 19.86 | 2.14 |
| 8 | 43.45 ± 10.86 | 120.05 ± 104.58 | 2.76 |

**Supplementary Table 8:** Felodipine plasma and brain concentration in minipigs. Analysis was performed after single dose of 2.5 mg/kg body weight orally (n=2 minipigs per time point). Data are presented as mean values ± SD.

**Supplementary Table 9: Felodipine plasma concentration in double transgenic mRFP-GFP-LC3/B6HD mice at steady level for 28 days**

| | Plasma conc. (ng/ml) | | |
|---|---|---|---|
| **mouse (n)** | **day10** | **day20** | **day28** |
| 1 | 8.15 | 18.4 | 18 |
| 2 | NS | NS | 17.1 |
| 3 | 7.24 | 16 | 9.05 |
| 4 | 12.2 | 9.1 | 29 |
| 5 | 18.8 | NS | 17.6 |
| 6 | 10.8 | NS | 5.67 |
| 7 | 26.7 | 36.6 | 29.6 |
| **mean** | **13.98 ± 2.82** | **20.03 ± 4.43** | **18.00 ± 3.41** |

**Supplementary Table 9:** Male mice were implanted subcutaneously with felodipine-loaded osmotic minipumps 5 mg/kg body weight/ day (Alzet Model 2004 with 0.25 µl/hr flow rate) for 28 days. Blood samples were collected at day 10, 20 and 28 (terminal) for analysis. Data is presented as actual values for individual mice and mean value +/- SEM is provided for each time point. Mice where the pumps showed reduced flow rate (i.e. plasma concentration < 5 ng/ml) and those with blocked pumps (determined by remaining volume) were excluded from analysis. NS indicates no blood sample was collected at that time point due to sampling problems.
