# Supplementary material for: Felodipine induces autophagy in mouse brains with pharmacokinetics amenable to repurposing
Source: Nat Commun. 2019 Apr 18;10:1817. doi: 10.1038/s41467-019-09494-2 (PMC6472390; doi:10.1038/s41467-019-09494-2)
Supplement: Supplementary file 2 — Peer Review File [file 41467_2019_9494_MOESM2_ESM.pdf]

Reviewers' comments:

Reviewer #1 (Remarks to the Author):

An impressive and highly significant study, which really pops the question on whether felodipine can be used of clinical use. It is rare our days to see such thorough, methodical, and well-controlled drug repurposing investigation with highly appropriate statistical analysis. Really well done!
Minor point on whether authors tried or plan to test the drug in full-length HD mouse model?
More important and critical point and I would appreciate author response.
It is apparent that after oral administration of felodipine in minipigs, brain concentration is about 20 times worse then after I.P. in mice. I am aware of comparison oranges and apples, as yet that is a concern for human drug. Does or would oral administration of felodipine mediate the efficacious effects in N171-82Q and SNCA mice?
Sincerely, Alex Kazantsev

Reviewer #2 (Remarks to the Author):

Siddiqi et al. report that felodipine, a L-type calcium channel blocker and anti-hypertensive drug can induce autophagy in primary neurons and clear aggregates of mutants of tau, Huntingtin and α-synuclein proteins in zebrafish and mouse models, respectively. Furthermore, treatment of felodipine improved the response of mice modeling Huntington's disease in a number of behavioral studies. The authors provide pharmacokinetic studies in mice and minipigs to show that pharmacodynamic effects in vivo correlate with drug levels and effects in the plasma and brain. Based on known drug levels of felodipine in human plasma the authors conclude that repurposing of felodipine for neurodegenerative diseases should be attainable.

This is a very interesting and novel study showing promising activity of felodipine as autophagy inducer in primary neurons for the treatment of neurodegenerative diseases. The authors have provided sufficient data to suggest that felodipine can reach appropriate drug levels in the brain of mice to induce autophagy and clearance of aggregates of proteins, which suggest felodipine repurposing.

Repurposing of felodipine would be successful only if felodipine can have similar CNS permeability in humans and reach the levels that are able to induce autophagy in neurons as in mice. The author's suggestion about "amenable to reperpusing" is based on human plasma levels of felodipine. The authors should provide any available information about brain levels of felodipine in humans or provide a discussion about this.

I have a few additional comments:

1. Figure 1 b: The quality of the images is poor, making it difficult for the reader to observe the phenotype. It will also be more helpful to have an additional zoomed panel showing the foci that were scored per condition. Why the scale bars among the panels have different size? Do the differences in the acquisition conditions can affect the measurements and the quantification?

2. Figure 1 f: Can the authors define the morphological defects? It is not clear how the morphology of the subjects was classified as normal, mild defective etc.

3. Figure 4C data are key to suggest that the felodipine works in low nM concentrations as would be expected in humans. Authors are encouraged to increase the number of experiments. Have the authors tested felodipine in similar concentrations in human iPS-derived neurons?

4. Figures 4D and E: the authors will need to provide representative examples of the imaging that is used to perform the quantifications.

Responses to reviewers

Reviewer 1:

An impressive and highly significant study, which really pops the question on whether felodipine can be used of clinical use. It is rare our days to see such thorough, methodical, and well-controlled drug repurposing investigation with highly appropriate statistical analysis. Really well done!
Minor point on whether authors tried or plan to test the drug in full-length HD mouse model?

Many thanks for the enthusiastic comments.

We have not studied the HD full-length models. These require much larger sample sizes and longer studies compared to the exon 1 model due to the slower and weaker phenotypes. Most importantly, these would not be suitable for the experiments aiming to test felodipine under conditions mimicking the human situation, as we did with the A53T alpha-synuclein mice. Such experiments require subcutaneous minipumps, and since we can only change them once, we only have a one month window for our experiments. We were fortunate that the A53T alpha-synuclein mice had a disease course that was amenable to this strategy – unfortunately, this is not the case with the full-length HD models. In due course, we may try to adapt the delivery strategy to be able to test full-length HD mice.

More important and critical point and I would appreciate author response.
It is apparent that after oral administration of felodipine in minipigs, brain concentration is about 20 times worse then after I.P. in mice. I am aware of comparison oranges and apples, as yet that is a concern for human drug. Does or would oral administration of felodipine mediate the efficacious effects in N171-82Q and SNCA mice?

If one compares table 3 (mouse I.P. PK data) and Table S8 (minipigs oral), then it is apparent that the plasma concentration in the mice peaks at 1151 ng/ml, while that in the minipigs in 76 ng/ml. Thus, the amount that gets into the blood is very different in the two species via different modes of administration. However, the concentrations are higher in the brain than in plasma in both species, which is the key point. A similar trend is seen in Table 4, which examines these parameters when the drug is administer subcutaneously with minipumps. Here the plasma concentrations are much lower, but again there is more in the brain (see 14 day data when plasma and brain data are comparable) – here the brain/plasma ratio is quite similar again to what we saw in the minipigs. So, the different amounts in the brain in the different experiments are likely a reflection of different amounts in the plasma (different levels administered).

Please see new text in penultimate paragraph on p14: "We are not aware of any data on felodipine brain concentrations in humans. Accordingly, we have assessed brain/plasma concentrations of the compound in mice under i.p. and subcutaneous minipump regimes in mice and via oral dosing in minipigs and find that felodipine concentrates in the brain, hence we would speculate that this is likely to be the case in humans."

Our data predict that if we devised an oral dosing regime that enabled blood concentrations of the drug that mimicked those seen in humans (as we achieved with subcutaneous minipumps), then this should be efficacious.

Reviewer #2 (Remarks to the Author):

This is a very interesting and novel study showing promising activity of felodipine as autophagy inducer in primary neurons for the treatment of neurodegenerative diseases. The authors have provided sufficient data to suggest that felodipine can reach appropriate drug levels in the brain of mice to induce autophagy and clearance of aggregates of proteins, which suggest felodipine repurposing.

Repurposing of felodipine would be successful only if felodipine can have similar CNS permeability in humans and reach the levels that are able to induce autophagy in neurons as in mice. The author's suggestion about "amenable to reperpusing" is based on human plasma levels of felodipine. The authors should provide any available information about brain levels of felodipine in humans or provide a discussion about this.

Many thanks for the enthusiastic comments.

We are not aware of any data on felodipine brain concentrations in humans. Accordingly, we have assessed brain/plasma concentrations of the compound in mice under I.P and subcutaneous minipump regimes in mice and via oral dosing in minipigs. All of these experiments (tables 2, 3, 4, 5, and S8) suggest that the felopipine concentrations in the brain are likely to be higher in brain than in plasma in humans.

We have added appropriate text to the discussion – see page 14 line penultimate paragraph:

"We are not aware of any data on felodipine brain concentrations in humans. Accordingly, we have assessed brain/plasma concentrations of the compound in mice under i.p. and subcutaneous minipump regimes in mice and via oral dosing in minipigs and find that felodipine concentrates in the brain, hence we would speculate that this is likely to be the case in humans."

I have a few additional comments:

1. Figure 1 b: The quality of the images is poor, making it difficult for the reader to observe the phenotype. It will also be more helpful to have an additional zoomed panel showing the foci that were scored per condition. Why the scale bars among the panels have different size? Do the differences in the acquisition conditions can affect the measurements and the quantification?

We have provided new images with identical scale bars in Fig S1A. The images were all acquired under identical conditions. Please note that the quality is low because we acquired the images via live cell microscopy to minimise any alterations in the red versus green fluorescence emission due to fixation.

2. Figure 1 f: Can the authors define the morphological defects? It is not clear how the morphology of the subjects was classified as normal, mild defective etc.

These were described in detail in Lopez et al (ref. 13). We have now added a schematic to Fig S2A to make the data interpretable without the need to refer to the previous paper.

3. Figure 4C data are key to suggest that the felodipine works in low nM concentrations as would be expected in humans. Authors are encouraged to increase the number of experiments. Have the authors tested felodipine in similar concentrations in human iPS-derived neurons?

Fig 4C was performed three times (biological replicates) in triplicate and significant effects were seen at all concentrations, two of which (50 nM and 100 nM are within the range we have inferred to be likely in human brains).

We have now added new data in human iPS-derived neurons. We had access iPS-devided neurons that were engineered to have a homozygous A53T alpha-synuclein mutation that allows us to measure A53T levels - this is useful as A53T is a very good autophagy substrate. A53T levels were reduced by 100nM felodipine (a concentration we think is likely in human brains). We have added these data in Fig 5A to complement the mouse experiments with this mutation.

4. Figures 4D and E: the authors will need to provide representative examples of the imaging that is used to perform the quantifications.

Please see examples in revised fig 4D and 4E.
